# UAV-based RGB and multispectral mango leaf disease detection with benchmarking of YOLOv5 to YOLOv10 and SeqOpt-optimised YOLOv8 for real-time edge deployment

**DOI:** 10.1371/journal.pone.0349855

**Published:** 2026-05-28

**Authors:** R. P. Karthik, G. Murugesan, Hattan Khaled Ballaji, J. Anitha

**Affiliations:** 1 Department of Electronics and Communication Engineering, Kongu Engineering College, Perundurai, Erode, Tamil Nadu, India; 2 Department of Computer and Network Engineering, College of Computing, Umm Al-Qura University, Makkah, Saudi Arabia; 3 Department of Electronics and Communication Engineering, Karunya University, Coimbatore, Tamil Nadu, India; Sultan Qaboos University, OMAN

## Abstract

The article presents a real-time mango leaf disease detection framework with embedded edge deployment, using UAV-based multispectral imaging combined with optimised deep learning models. A custom dataset of 6,334 high-resolution RGB and multispectral images representing four common diseases was collected under natural orchard conditions using MAPIR RGB and multispectral OCN cameras mounted on a UAV. A controlled benchmarking of YOLOv5-YOLOv10 architectures was performed under identical training configurations. Although YOLOv10 achieved the highest detection accuracy, YOLOv8 offered a more favourable balance between detection performance and deployment efficiency on edge devices. To further enhance robustness and deployment suitability, the proposed SeqOpt method was applied to YOLOv8, improving the F1-score by 9.7%, mAP@50 by 8.6%, and mAP@50–95 by 21.7% compared to YOLOv10 trained under identical conditions on the multispectral validation data. In addition, on the Raspberry Pi 5 using ONNX inference, latency and energy consumption were reduced by up to 25% compared to the SGD-only YOLOv8 baseline. On the NVIDIA Jetson Orin Nano, the PyTorch model achieved 72 ms per-image inference latency, demonstrating near real-time capability. Overall, the proposed pipeline outperforms single-optimiser YOLOv8 (SGD-only and AdamW-only) and YOLOv10 baselines in detection accuracy and deployment efficiency, making it suitable for practical precision agriculture applications.

## Introduction

The Mango (Mangifera indica), commonly referred to as the “King of Fruits” originated in Southeast Asia and remains one of the most economically significant tropical fruit crops worldwide. Despite its economic and nutritional significance, large-scale production faces significant challenges including pest infections, fungal and bacterial diseases, high labour cost, and difficulty in early disease detection. All these contribute to a huge decrease in the yield of crops affecting both small- and large-scale producers. Disease monitoring and early detection have become important areas among the different fields of precision agriculture. The integration of remote sensing and deep learning provides a non-invasive and scalable approach to monitor crop health and detect disease symptoms at early stages, before visible damage becomes widespread [1–3].

Mango trees are susceptible to several diseases, including anthracnose (*Colletotrichum Gloeosporioides*), dieback (*Botryosphaeria Ribis*), sooty mould (*Meliola Mangifera*) and bacterial canker (*Pseudomonas Syringae*, *Pseudomonas Morsprunorum*). These diseases are capable of reducing fruit quantity and quality. The traditional method of disease inspection is time-consuming, expensive, and cannot be applied in large orchards. The present paper introduces a UAV (Unmanned Aerial Vehicle)-based mango leaf disease detection system based on both RGB (Red, Green, Blue) and multispectral images. This study involved the construction of a custom drone with a MAPIR Survey 3W multispectral OCN (Orange, Cyan, NIR-Near Infrared) camera to overcome the shortcomings of the traditional disease inspection techniques. As opposed to online datasets, which are usually not diverse enough to provide accurate disease detection, real-world datasets were gathered across diverse environments and orchard conditions. This ensures that the data used in this reflects real variability in the mango orchards. This study framework will provide benchmarking of the YOLO (You Only Look Once) [4–6] family of object detection models (YOLOv5 to YOLOv10) to detect spectral-based mango disease. Beyond benchmarking, YOLOv8 emerged as the most balanced architecture in terms of detection accuracy, inference speed, and architectural efficiency, making it well-suited for field deployment embedded systems.

To further improve real-time performance, SeqOpt (Sequential SGD-AdamW optimiser), a sequential optimization strategy that uses SGD (Stochastic Gradient Descent) with AdamW (Adaptive Moment Estimation with Weight Decay) was proposed. This approach enhances the convergence and the model generalisation. Moreover, YOLOv8SO was exported to both ONNX (Open Neural Network Exchange) and PyTorch formats. Both formats were deployed and evaluated on two edge devices, Raspberry Pi 5 and NVIDIA Jetson Orin Nano. Speed of inference, memory consumption, model effectiveness, and power consumption were compared at 1024 x 1024 resolutions in actual orchard. The study is aimed at achieving three objectives that are interconnected. First, it presents a comparative analysis of YOLO architectures to classify mango leaf diseases with the help of spectral image data. Second, this study evaluates the deployment of the proposed models on edge devices – NVIDIA Jetson Orin Nano and Raspberry Pi 5, for real-time inference in a farm field condition. Lastly, it suggests an intelligent decision-support system that can be scaled to enhance disease management procedures to increase productivity in mango production. The key aim and contribution of this works are summarised in following section below.

In addition, this paper measures the performance of real-time deployment using standard detection and efficiency metrics, as detailed in the results and discussion section. These parameters make sure that the models operate efficiently in the real-world in the agricultural environment. The proposed research provides a powerful, field-deployable system of mango leaf disease detection through the combination of RGB and multispectral image processing with deployment-ready machine learning models. In contrast to traditional systems, the proposed framework enables detection of multiple disease classes with low latency, directly on embedded hardware in orchard settings.

### Aim and contributions

i The benchmarking of YOLOv5 to YOLOv10 was conducted under strictly identical training, testing, and preprocessing environments. The dataset comprised field-collected UAV imagery of four mango leaf disease classes from in real orchard settings, in both RGB and OCN multispectral modalities.
ii Systematic study of optimiser behaviour across YOLO variants resulted in SeqOpt – a fixed-checkpoint schedule with no architectural modifications. It combines SGD in the early phase for stable convergence, followed by AdamW in the final phase.
iii All variations of YOLO were evaluated on embedded hardware to measure the inference latency, runtime stability and per-image energy consumption. This enables direct comparison of detection accuracy and deployment cost in resource-constrained systems.
iv The optimised multispectral YOLOv8 model was evaluated on both a CPU-only platform (Raspberry Pi 5) and a GPU-enabled platform (NVIDIA Jetson Orin Nano). This provides a practical understanding of model behaviour across heterogeneous UAV-deployable edge devices.

## Related work

The earlier research attempts of mango disease detection largely depended on the use of RGB images that had been obtained under controlled conditions, and hence, could not be generalised to the conditions in the real-world. The multi-modal approaches are based on the extensive review by Upadhyay et al.[7], who differentiated the models based on architecture, data diversity, and crop of interest. Abudukelimu et al. [4] introduced DM-YOLO which is a variant of the YOLOv9 that is designed to add loss functions and sampling techniques to improve the precision of detecting small objects which is used in tomato leaf disease detection. Mahmud et al.[8] created a light CNN (Convolutional Neural Network) -based model that is optimised to deploy edges in mango leaf classification; the model is lightweight, and inference time is largely reduced, as well as the performance is not compromised. Equally, Gautam et al. [9] proposed the use of an ensemble stacked deep neural network, which has high mAP (mean Average Precision) scores on real-world datasets. Other methods which are getting momentum are cloud integration and transfer learning. Lanjewar et al [10] capitalised on mobile PaaS platforms to detect citrus leaf disease using the integration of data augmentation and CNNs with the cloud computing.

Banerjee et al. [11] used CNN and SVM (Support Vector Machine) model to classify mango disease in terms of severity showing enhanced robustness as compared to the traditional model. Admass et al. [12] used a hybrid method and combined both HOG (Histogram Oriented Gradients) and CNN characteristics to achieve a better mango leaf classification. Velasquez C et al. [13] used the visible and near infrared hyperspectral imaging and linear discriminant analysis to enhance the detection of anthracnose in mango fruits at the early stage and enhanced the accuracy of the process. MangoLeafBD, a comprehensive annotated dataset of large size designed by Ahmed et al. [14] can be used to train models and benchmark them. As observed in Shafik et al. [15] transfer learning has been vastly implemented, which has done well in the area of generalisation to unseen plant diseases. Haridasan et al. [16] used CNN and SVM fusion in the classification of rice disease and the results would have 0.91% in various disease categories. At the same time, Tsuchikawa et al. [17] investigated the NIR spectroscopy and Liu J et al. [18] portable quality assessment model to improve the monitoring of forests and crops. Wani et al. [19] provided a methodological review of the applications of Machine Learning and Deep Learning to detect plant diseases and highlighted the importance of transfer learning and explainability. Finally, Sanath Rao et al. [20] presented an effective CNN-based transfer learning architecture to predict grape and mango diseases and optimised to work with mobile devices and edge computing.

Although previous research work has shown that deep learning models can be used for accurate plant and mango disease detection using RGB, multispectral, or hyperspectral images, there are still some gaps in the current literature. Most of the previous research work has focused on a single deep learning model or a single type of optimiser, and very few research works have analysed the performance of recent versions of the YOLO algorithm under equal training and data conditions. There is very little research work available on the behaviour of optimisers and the convergence properties of multiple optimisers, especially in multispectral images. In addition, most of the previous research work has focused on the performance analysis of models in offline or GPU-based scenarios, and very few research works have focused on real-time viability, energy efficiency, or real-time stability in low-power edge devices.

### Study area

**Fig 1:**
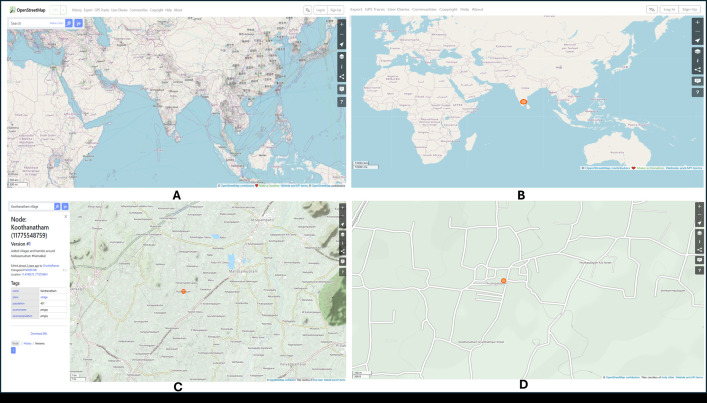
Location of the experimental study area at multiple spatial scales. (A) regional context, (B) country-level view, (C) district-level view, and (D) local site view. Map data © OpenStreetMap‌‌ contributors, available under the Open Database License.

The research process includes monitoring the infected mango trees by the diseases and the datasets were collected in the mango orchard in the Koothanatham village, Mallasamudram town, Namakkal district, Tamil Nadu, India. It is considered a hot, semiarid ecoregion which thrives on red loamy soil; the soil is suitable in the growth of mango. Therefore, the mango orchard at this area was selected to be used in the experimental study. **Fig 1** represents the plot area of experimentation, wherein, the canal and tube well irrigation techniques were employed.

## Materials and methods

**Fig 2:**
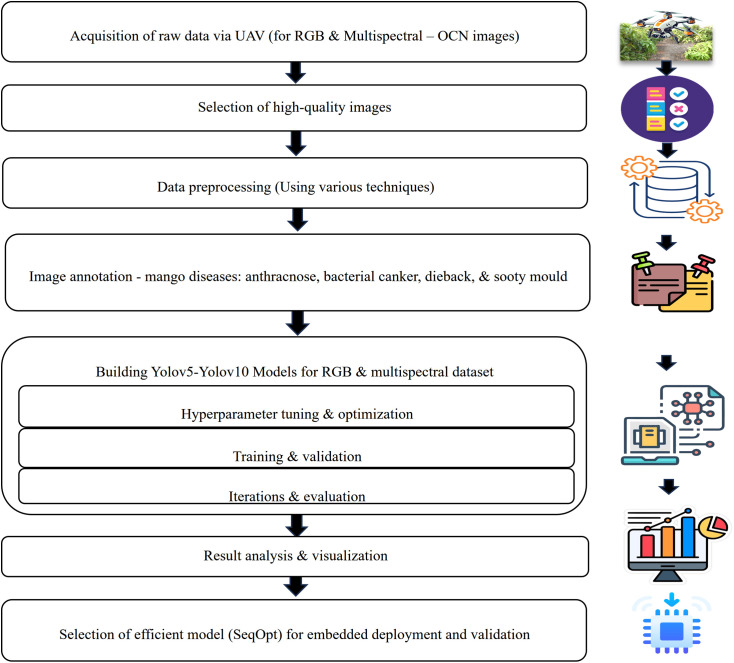
Overview of the proposed research workflow for UAV-based mango leaf disease detection. The workflow includes UAV image acquisition, dataset preparation for RGB and multispectral (OCN) modalities, model training and benchmarking across YOLO variants, Sequential SGD-AdamW (SeqOpt) optimisation, and deployment evaluation on edge platforms.

The identified approach to the problem is the identification of the best deep learning algorithms to detect and classify mango leaf disease in real-time with the help of embedded edge-AI devices (e.g., NVIDIA Jetson, Raspberry Pi 5) to deployed UAVs. This study benchmarks YOLOv5-YOLOv10 to identify the most suitable model for edge deployment. To achieve high-quality stable real-time image data, a custom drone with an RGB, multispectral camera and embedded computing system was designed and deployed. The stages of the workflow are drone design with mitigation measures, preparation of specific dataset (data annotation and preprocessing), hyperparameter optimisation on embedded hardware, and the selection of an appropriate deployment model. The method takes advantage of multispectral and RGB images taken with a MAPIR Survey 3W camera, which allows identifying diseases precisely. Real-time decision-making can be enhanced with the application of high-resolution image and edge AI, which facilitates the more effective management of the diseases in mango plantations. The major flow of the proposed work is shown in **Fig 2**.

### Acquisition of an image with a customised drone

The mango leaf diseases are efficiently reported by the deployment of a customised drone with a the MAPIR Survey 3W camera. It has multispectral and RGB imaging with the use of this camera, which facilitates the acquisition of high-resolution and real-time data. The drone is operated manually as it takes a predefined flight route around the mango orchards to cover all areas with effective coverage and homogeneous data collection. The altitude and speed are optimised to achieve resolution and coverage and take images in the most favourable lighting conditions in order to improve the quality of the data collected [21]. Achieving optimal performance of the inertial measurement unit (IMU) and mitigating electromagnetic interference from the power distribution board (PDB) required careful design and integration during drone construction. Moreover, the 2D gimbal is provided in order to have a more stable camera. The difficulties, solutions, and customisation approaches that were adhered to during the drone design and development stage are summarised below.

### Mitigation strategies‌‌

**Fig 3:**
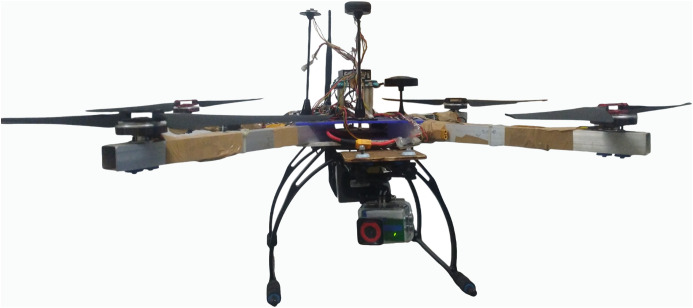
Custom UAV platform used for data acquisition, equipped with a MAPIR Survey 3W multispectral camera and associated payloads. The setup was used to capture RGB and multispectral (OCN) imagery under real-field conditions for dataset creation.

To stabilise voltage and filter noise, high quality capacitors close to the IMU input are used with the help of an isolated low-noise power supply to eliminate system interference. Star grounding scheme reduces the amount of impedance and eliminates ground loops, which ensures that there is a common low-noise ground between the PDB and the IMU. In the IMU, EMI is blocked by shielding with a metallic casing that has low weight. To minimize mechanical vibration disturbances the IMU and switching power regulators are physically separated and linked with vibration-damping mounts. Signal and power line low-pass filter block the switching noise at high frequencies. Lastly a strong, tuned IMU is employed and PCB layout segregates IMU tracks with noisy high current tracks [22]. This proposed work employs a customised UAV platform image acquisition as illustrated in **Fig 3**.

### Drone equipped with multispectral and RGB cameras

The use of specialised imaging devices such as multispectral cameras can allow to detect the problems with the health of the plant at an early stage because they record data in a variety of spectral bands. In this research, the MAPIR Survey 3W multispectral OCN (Orange – 615 nm, Cyan – 490 nm, NIR – 808 nm) camera was used together with an RGB (visible light – 375 nm to 650 nm) camera in the capture of high-resolution images of the mango orchards. Survey 3W camera is a 3.37 mm focal (**Table 1**) length camera that records spectral data that is important in detecting diseases and stress symptoms including chlorosis, necrosis and cellular degradation. The OCN bands have better spectral sensitivity compared to the RGB cameras, which are only sensitive to visible light.

**Table 1:** Technical specifications of the MAPIR Survey 3W camera system used for data acquisition.

S. No.	Specifications	Range
1.	Photo Resolution	12MP (4,000 X 3,000)
2.	Image Format	RAW (12 bit) + JPG (24 bit), JPG (24 bit)
3.	Sensor	Sony Exmor IMX117 12MP (Bayer RGB)
4.	Chipset	Novatek NTK96663
5.	Photo Interval	0.5s (JPG), 2.8s (RAW + JPG)
6.	Shutter Speed	1/2000–1 min, Auto
7.	Storage	MicroSD (up to 128GB)
8.	Radio/Sensor	Wi-Fi, Bluetooth
9.	Battery	1200mAh Li-Ion (150 mins)

The changes in pigmentation [19] of the leaves are detected by the orange band (590–620 nm) because of fungus or bacterial infections, and the cyan band (490–520 nm) indicates the early signs of stress caused by nutritional deficiency or drought. The NIR band (750–900 nm) detects the internal damaging effects of the leaves by recording the changes in light reflectance, as the healthy leaves reflect more of the NIR light than the diseased ones. A drone which had MAPIR cameras was used to record images at a height of 5–20 metres and 3–10 m/s, which provided a balance between resolution and coverage. To have an efficient processing of 4K, the images were down sampled to 1K to reach a GSD of 0.091 to 0.362 inches/pixel. **Table 1** represents the specifications of the MAPIR Survey 3W model camera used for this study.

### Data preprocessing

**Fig 4:**
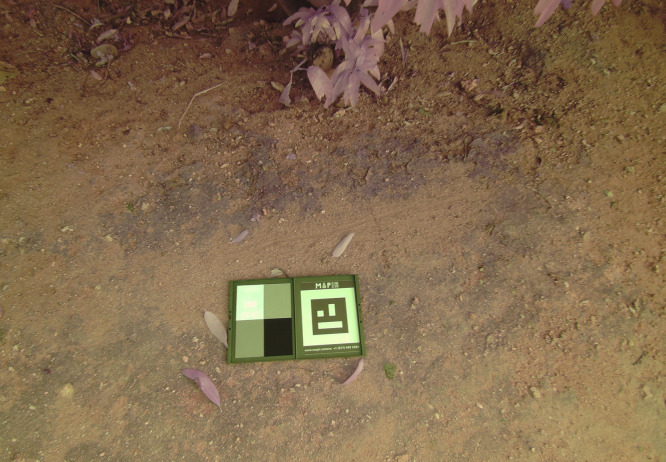
Calibration target image captured by the UAV-mounted camera during pre-flight calibration. The calibration target used to ensure accurate radiometric and geometric image acquisition.

The pre-processing of data is essential in proper mango leaf disease detection with the help of drone imagery [23]. It meets the needs in relation to lighting differences, noises, improving the clarity of images, and standardisation of data. The white balance correction enhances the accuracy of colours whereas noise removal retains important features. Data augmentation enhances the diversity in the datasets, and this allows the models to generalise. The steps increase the precision, strength, and the real-time of deep learning models in identifying diseases. **Fig 4** illustrates a calibration target that has been captured by the to a camera to correct the effects of colour and UV lights. MAPIR Camera Control (MCC) was used to process the calibration target images to carry out radiometric and UV correction which helps to reduce light bleeding and associated artifacts in both the multispectral and RGB images.

### White balance adjustment

The colour balance of raw images produced by the MAPIR Survey 3W camera is usually affected by the variation of the lighting conditions including the changes in the sunlight intensity and angle. White balance adjustment equalises colours and makes sure that the RGB and OCN channels are represented accurately. The step is essential in enhancing the accuracy of feature detection particularly on the differentiation of healthy and diseased leaves because minute colour changes can be used to predict certain diseases.

### Data augmentation

Flipping, rotation, scaling, cropping, brightness, mix-up, and spectral transformations, which are examples of data augmentation, increase the diversity of the dataset and model resilience to real-world variations [24]. These extensions aid higher generalization of the YOLO models to detect mango leaf disease. The parameters of noise, hue, saturation and exposure augmentation were adjusted up to 1% percent, 20% and 10% respectively.

Stereotypical YOLO augmentation tactics were employed equally to all variants of models (YOLOv5 to YOLOv10) to allow a fair and consistent comparison in conditions of UAV-based image acquisition.

### Noise removal

The sensor limitations or environmental conditions make drone imagery noisy, which is reduced with the help of Gaussian and median filtering, and important leaf characteristics are retained. Multispectral noise reduction is achieved by sensor calibration with reference reflectance values which are determined by fixed colour correlation as had been mentioned above. The correction of UV and colour is done with specialised hardware (**Fig 4**) and software (MCC). These preprocessing mechanisms increase the quality of the images and show disease-specific characteristics which allow higher detection and classification [25].

### Dataset creation and balancing

#### Division of disease classes.

**Fig 5:**
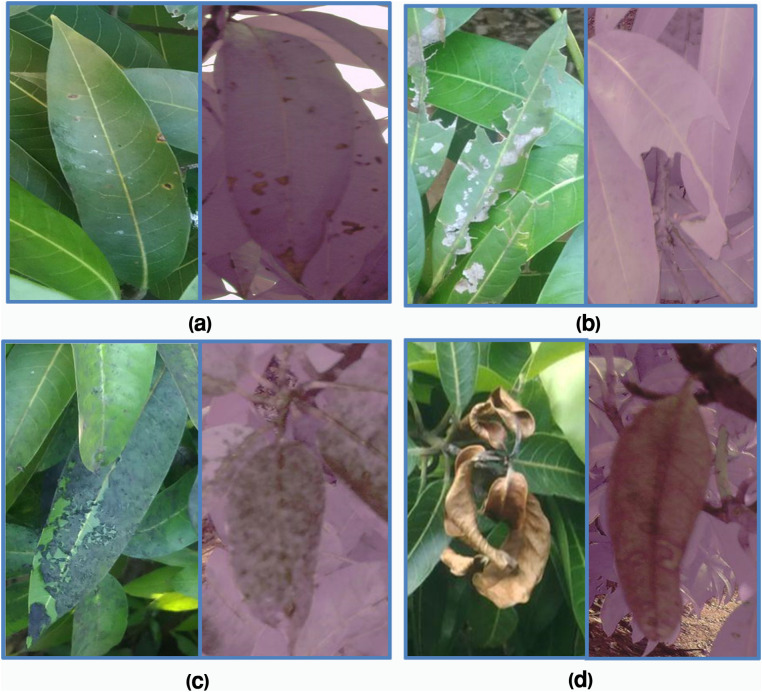
Representative annotated images illustrating the four mango leaf disease classes used in this study. The figure illustrates the four disease categories: (a) anthracnose, (b) bacterial canker, (c) sooty mould, and (d) dieback. (RGB-left side, multispectral-right side)**.**

First, several types of diseases were considered to be screened and analysed. But in the process of collecting data, some of the classes were discovered to have small numbers of samples in the study area such as powdery mildew. Due to that, balancing the data set with either data augmentation or synthetic would not be [10,24] feasible or meaningful. Therefore, the model was narrowed down to concentrate on the four main and enough available types of disease, which were anthracnose, bacterial canker, sooty mould and dieback. **Fig 5** represents a sample RGB and multispectral images of the four categories of diseases that were captured and annotated during the study.

#### Dataset annotation and partitioning.

A properly labelled and balanced dataset is indispensable to the efficient real-time mango leaf disease detection. LabelMe and Roboflow software annotation tools are used to label high-quality and relevant images. The annotations of objects were spatially various in the whole image frame, and tight rectangular bounding boxes enhanced the accuracy of edges. Such an improved model strength facilitates the correct object detection irrespective of object location in real life environment. The data was split into training and validation sets with a fixed split strategy of 70% for training and 30% for validation, where the validation set was used for evaluation of the models during experimentation.

#### Image quality control and image annotation.

Bounding boxes were used to mark an annotation on the disease-affected areas on mango leaves, and the annotations were exported in the YOLO-compatible text format. The preparation of annotations was done manually in accordance with the protocol of labelling as below mentioned. Before annotating, images with an extreme motion blur, high visibility, or lack of visual detail were eliminated. The annotated labels were checked on consistency and completeness of the classes and then they were finalized to train the model.

Bounding boxes were placed carefully in the course of annotation to ensure that neighbouring objects were not overlapped as well as uniformity was maintained in the scale of objects. Very small or unclear areas that could not be distinguished reliably were eliminated in order to eliminate noisy labels. In order to further guarantee consistency in annotations, a custom python-written validation script was employed to perform preprocessing of the potential problems that may have occurred like malformed or unintended bounding boxes before model training.

#### Prevention of information leakage.

Data dividing into various subsets for limiting possible information leakage was done based on UAV flight sequences, instead of dividing based on individual images. The photos of the same flight session were indexed into one dataset partition. This was to make sure that similar looking frames are never divided in training and validation sets. Pictures that were blurred or the ones that could not be identified with poor visibility were discarded prior to processing or segregating datasets based on image characteristics. Thus, each model was trained and tested on equally split data sets to be consistent in the application of visually distinguishable and non-overlapping samples by models and minimize any additional sources of information leakage.

#### Dataset statistics table.

The composition of the datasets of the UAV-based mango leaf diseases used in this study is summarized in **Table 2**. The dataset (6,334 images) is made up of two independent modalities RGB and multispectral (OCN). The RGB subset is composed of 3,605 images (2,628 training and 977 validation) and the multispectral subset is composed of 2,729 images (1,911 training and 818 validation). Four classes of diseases have been annotated under each modality (anthracnose, bacterial canker, dieback, and sooty mould). In total, the RGB subset consists of 42,587 annotated instances whereas the multispectral subset consists of 36,673 annotated instances. While RGB and multispectral images were derived independently and uncoupled at an individual leaf level, with a common annotation scheme, these data are paired. All YOLO models (v5 to v10) were trained and tested with the same data split strategies so that it is possible to make a fair comparison. In the current study, the validation part was used for model evaluation and comparison in all the YOLO variants. The same training and validation splits were used each time during deployment stage also, so that it is compared fairly.

**Table 2:** Summary of the UAV-based mango leaf disease dataset used in this study.

Modality	Total Images	Train Images	Validation Images	Anthracnose	Bacterial Canker	Dieback	Sooty Mould
**Multispectral (OCN)**	2,729	1,911	818	12,295	10,696	2,921	10,761
	Balanced data resampling values			1	1.1495	4.2092	1.1426
**RGB**	3,605	2,628	977	9,812	15,924	1,602	15,249
	Balanced data resampling values			1.6229	1	9.9401	1.0443

#### Dataset balancing.

To avoid underfitting and overfitting and to guarantee the generalisability of the model, the dataset should be balanced with regard to the instances of classes, a number of images, training, validation, and testing datasets. The number of instances of disease in all the classes is not equal in proposed dataset, with some diseases like dieback having relatively fewer samples. The balanced data resampling method was employed (**Table 2**), which have assisted in balancing the number of instances in each of the classes leading to a more homogeneous dataset on the training, validation, and testing splits [26].

### Training consistency

All the YOLO models (YOLOv5 to YOLOv10) were trained with the same core training settings, which include the input image resolution, batch size, training epochs, data augmentation mode, training hardware, core allocation on the GPU, and clock setup. Architectural variations which were inherent to the individual YOLO versions were preserved. All the other hyperparameters and training conditions were held constant across models, with the exception of the optimiser scheduling proposed by the proposed SeqOpt strategy (applied to YOLOv8). The SeqOpt strategy used a rigid schedule on the transition of optimisers without hyperparameter optimisation of models, or architecture-specific optimisation. Therefore, the difference in performance is likely to be due to optimiser scheduling strategy and inherently designed models and not due to favourable hyperparameter selection.

### Architecture analysis of YOLO

YOLO family of real-time object detection models (YOLOv5 -YOLOv10) was used in this research because the single-stage detection can be applied and it has been suitable in time-sensitive agricultural settings [4,23,27]. Each of the models was unchanged and utilized as it was presented by the Ultralytics framework. In order to have a fair and consistent comparison across versions and to be able to run them on resource-constrained edge devices, the small (S) versions of all YOLO models were only tested. The variation in performance across YOLO versions thus reflects their underlying architectural designs with the emphasis of the study being on training strategy, optimisation behaviours and deployment performance and not on architectural reengineering.

### Disease detection framework

The mango leaf disease detection model in the paper is applied to the YOLO object detector framework to detect and localize the symptoms of a disease in the UAV-taken images. The framework is set up to identify various classes of diseases which include anthracnose, sooty mould, bacterial canker and dieback through learning visual features in terms of shape, colour and texture. The default YOLO detection pipeline was applied without any modification of the underlying architecture, which enabled the model to work effectively and offer real-time inference on the resource-limited agricultural surveying settings [28].

### Hyperparameter selection

The aspect of hyperparameters is important in the performance and generalisability of the deep learning model. They are also not trainable as they are confided before training and directly affect the convergence, accuracy and stability of models. The most important hyperparameters are the learning rate, the batch size, the type of optimiser, the image resolution, the number of training epochs, and the methods of regularisation. The default parameter settings yielded a range of 70–82%, 55–75%&, 70–82%, and 45–60% as the recall, precision, F1-score, and mAP@50, respectively, which requires optimisation. Hyperparameter optimisation is a method to overcome these drawbacks, which involves adapting the model to the data, improving the learning rate and its detection rate [29].

An experiment based on a manual grid search was conducted in order to find the optimal values of hyperparameters and the following ranges of candidates were evaluated: learning rate (0.01, 0.005, 0.001), momentum (0.9, 0.905, 0.937), weight decay (0.0005, 0.001, 0.005), batch size (8, 16, 32) and input resolution (640x640, 1024x1024). The fact that more than hundred independent runs using different random seeds gave consistent results indicated that the adopted configuration was stable and not seed-dependent. Other hyperparameters were also left to the default settings of Ultralytics framework and were not changed in all the versions of YOLO (v5-v10). The last values were learning rate 0.001, momentum 0.905, weight decay 0.005, batch size 16, resolution 1024x1024, which were kept constant across all variants of the model. There was no automated tuning, e.g., Bayesian optimisation or neural architecture search. In this study, all experiments were used the small (S) variants of the YOLO models to ensure consistency in the comparisons. To enable the deployment of the models at the low-powered edge devices; the larger (L/XL) and smaller (Tiny/Nano) variants were not considered.

### Utilisation of optimisers

An optimiser plays a vital role as it helps to adjust the parameters in the training of YOLO models to ensure that the loss is minimised and the accuracy is increased. To detect the mango leaf disease, a variety of optimisers were experimented with on YOLOv5 to YOLOv10 to detect the disease. The initial training was done on SGD over YOLOv5 to YOLOv10 to obtain a performance baseline. YOLOv8 was found to be the most balanced to mango leaf disease detection. To improve learning further, the SeqOpt (Sequential SGD-AdamW Optimiser) which combines the properties of SGD with the adaptive updates of AdamW, and the effective weight decay was proposed. This combination method was much more effective in revealing the slightest symptoms such as lesions and discolouration conditions when UAVs were used. The detailed results to justify this improvement can be found in the results section [30].

### Sequential SGD-AdamW (SeqOpt)

Based on the optimiser exploration phase (systematic optimiser testing and evaluation), SGD and AdamW optimiser always showed favourable convergence behaviour when compared with other optimisers, i.e., Adam (Adaptive Moment Estimation), Radam (Rectified Adaptive Moment Estimation), Nadam (Nesterov-accelerated Adaptive Moment Estimation), RMSProp (Root Mean Square Propagation), Adamax (Adaptive Moment Estimation with Infinity Norm), AdaBound (Adaptive Bound Optimization), and auto (mode) optimiser provided in the framework. While SGD showed stable behaviour of early stage of convergence, AdamW showed improved fine-tuning behaviour, in later epochs. These complementary characteristics were the motivating force for the proposed Sequential SGD-AdamW (SeqOpt) optimisation strategy.

**Fig 6:**
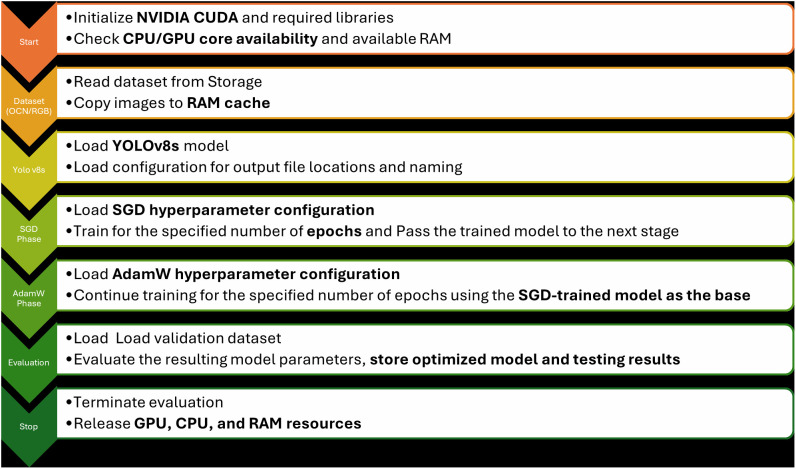
Overview of the proposed Sequential SGD-AdamW (SeqOpt) training workflow. The model is first trained using SGD for initial convergence, followed by AdamW for fine-tuning, before final evaluation on the validation dataset.

To build the SeqOpt model, the training was done in two successive stages as represented in the **Fig 6**, using the specified training parameter values. In the first phase, YOLOv8 was trained for 300 epochs with stochastic gradient descent (SGD) method with learning rate of 0.001, momentum 0.905, and weight decay 0.005. In phase two, training was then continued from the same checkpoint for 300 epochs with the help of AdamW with beta1 (0.9) and beta2 (0.999) parameters, and with weight decay 0.0005.

### Combined hyperparameter setup

The fine-tuning of several hyperparameters was done in unison in order to fit YOLO models to high-precision detection of mango leaf diseases in real-world agricultural. Many trial-and error approaches were undertaken to come up with the best values as indicated below. The resolutions of the images with 64x64 to 2560x2560 were investigated. Increased resolutions allowed more sophisticated patterns of disease to be determined at the cost of more computation. In the majority of experiments a compromise was reached in terms of detail and efficiency at 640x640 and 1024x1024.

A lower value of learning rate (about 0.005) compared to typical YOLO settings was used to stabilise the learning process and capture finer details of a disease in different lighting and texture conditions. Modifications in the momentum were made in order to increase the convergence speed without falling into the local minima as would normally take place in noisy farmers data. The early stopping was triggered by custom patience value, minimising the risk of overfitting by stopping the training when the validation loss did not decrease anymore. This is particularly useful in dataset that is naturally variable and has environmental artefacts. They had four data loader workers to ensure efficiency in training.

This environment provided the flow of data without overloading the CPU resources, particularly when augmenting the images and preprocessing them. The augmentation pipeline that YOLO uses was supplemented with custom based methods such as colour jittering, rotation, scaling, and noise injection. Such customised augmentation recreates environmental factors such as the orientation of leaves, disease, and natural lighting, among other factors, which greatly improves generalisation. Finally, the deep feature learning of the difficult UAV data was possible due to the optimal epochs of training. Although the count has surpassed the conventional limits, early stopping and monitoring helped to prevent overfitting that allowed real-time detection on edge devices to be robust.

### Ethics statement

The objective of this research project was to collect data using a UAV in a real-world mango orchard setting. Prior to collecting data, landowners gave permission for the research team to use their properties for UAV operation and research purposes. There were no human participants or animals included in this study; thus, no institutional ethics approval was necessary. UAV flights and all ground-based activities were conducted in accordance with relevant local law and established standard operating procedures (SOP).

## Results and discussions

In this section, the experimental findings of mango leaf disease detection across variety of YOLO models, including model benchmarking, optimiser behaviour, and real-time deployments on embedded edge devices. The accuracy of detection, computing efficiency and their suitability in the real-world are compared between RGB and multispectral UAV datasets.

### Experimental setup

YOLO models that were employed to detect mango leaf disease were trained with an input resolution of 1024 x 1024 to preserve the fine details and reduce the misclassification. This resolution choice was made in order to maximize accuracy while maintaining low computational cost for real-time UAV-based application with variable light and scale. The SGD was used as the optimiser in order to have reliability on real-time deployment, and they were trained with a batch size of 16, learning rate of 0.001 and momentum of 0.905. Techniques used to enhance generalisability included data augmentation and early stopping (patience = 10) was used to prevent overfitting during the models training of 300 epochs. The same hyperparameter values were used throughout YOLOv5 to YOLOv10 models.

The training was performed on a Lenovo ThinkSystem SR650 V3 (**Table 3**) server using an NVIDIA L4 server with 24GB of GPU-RAM and an Intel Xeon Gold 6430 processor. The data is stored in Samsung PM893 series SATA SD hard drive. **Table 3** displays the detailed GPU and CPU specifications. As a final experiment on the real-time edge deployment and testing, a Raspberry Pi 5 with an 8 GB RAM and NVIDIA Jetson Orin Nano platform with 8 GB RAM were used.

**Table 3:** Hardware specifications of GPU and CPU platforms used for model training and evaluation.

*GPU Specifications*		*CPU Specifications*	
Cores And Parameters	Nvidia L4	Item	Specifications
CUDA Cores	7,680	Processor	Genuine Intel 63
Tensor Cores	240	Model Name	Intel(R) Xeon(R) Gold 6430
RT Cores	60	CPU Cores	32
Memory	24 Gb GDDR6	CPU Clock	2,100 MHz
Memory Bandwidth	Up to 300 Gb/S	RAM	128 GB out of 256 GB
Max Power Consumption	72 W		

To account for variability and ensure reliable performance evaluation, the run strategies used at different stages of the experiment were varied. The final edge deployment testing of NVIDIA Jetson Orin Nano and Raspberry Pi 5 was done across 3 independent runs over inference, and the values that were reported are averages of the runs. The comparisons between optimisers were repeated twice or thrice to ensure that there are the same trends of performance. Benchmarking experiments YOLO training with RGB or multispectral were done once, since previous exploratory experiments always showed stable trends. Also, hyperparameter search has been done through manual grid search, as opposed to automated search; Bayesian or evolutionary optimisation can give an additional performance improvement. In cases where single runs are used to obtain results, it is clearly indicated and regarded as a limitation of the study.

### Parameter metrics overview

In order to evaluate the efficiency of the models of mango leaf disease detection, custom datasets (6,334 images), consisting of 3,605 RGB and 2,729 (**Table 2**) multispectral images were employed. Measures used in evaluation were precision, recall, F1-score, accuracy, mean-Average Precision (mAP@50, mAP@50–95), loss, inference time and error rates. Accuracy is the general correctness, but it might not be the true performance with imbalanced datasets. Precision also takes false positives, and recall takes false negatives and F1-score is a balance measure of detection. The mAP@50–95 also assesses the quality of detection at a number of thresholds. Multispectral imagery always excelled over RGB with its higher sensitivity to wavelengths and in the detection of malignancy at an early stage. These findings confirm the soundness of the model and its generalisability to various disease conditions.

### Optimisers performance

Out of the tested optimisers, SGD and AdamW (**Table 7**) have recorded positive convergence behaviour and classification rates. SGD demonstrated converged values that were robust and stable, low memory needs as well as predictable training dynamics; thus, SGD was suitable in real-time and resource-constrained deployment settings. AdamW, on the other hand, obtained better recall and F1-scores, especially in the symptoms of the disease at an early stage, which suggests a better ability to fine-tune at later training stages. Nevertheless, AdamW had a relatively large computational overhead, which may also impact the performance of real-time deployment when deployed independently. These complementary features inspired the application of sequential optimisation strategy. Specifically, the stability of SGD is used in the initial stages of training, while AdamW is applied in the later stages to refine convergence.

### Comparison of results analysis YOLO models

#### Critical examination of the metrics of YOLO models.

A full comparison of the YOLOv5 and YOLOv10 on RGB and multispectral datasets, including precision, recall, accuracy, F1-score, mAP, losses, and errors is summarised in **Table 4**. The object detection mentioned above was addressed in more detail in order to test the capabilities of real-time implementation on the embedded platform.

**Table 4:** Detection performance, loss behaviour, and model complexity metrics for YOLOv5-YOLOv10 evaluated on RGB and multispectral datasets.

YOLO Version	Data Type	F1 Score	Prec.	Rec.	Acc.	mAP@ 50	mAP@ 50-95	Box Loss	Class Loss	DFL Loss	MSE	MAE
YOLOv5	R	**0.832**	**0.911**	**0.765**	0.894	**0.845**	**0.65**	1.001	0.721	1.013	0.016	0.113
	M	0.806	**0.871**	0.75	0.888	0.825	**0.613**	0.998	0.734	1.057	0.014	0.106
YOLOv6	R	0.88	**0.949**	**0.82**	**0.949**	0.816	0.63	–	–	–	**0.003**	**0.051**
	M	**0.88**	**0.938**	**0.827**	**0.946**	**0.833**	0.601	–	–	–	**0.003**	**0.054**
YOLOv7	R	0.742	0.816	0.68	0.896	0.742	0.405	**0.051**	**0.003**	**0.046**	0.013	0.104
	M	0.713	0.809	0.638	0.887	0.729	0.363	**0.058**	**0.007**	**0.083**	0.015	0.113
YOLOv8	R	**0.833**	**0.897**	**0.777**	0.899	**0.856**	**0.653**	**0.929**	**0.634**	**0.983**	0.013	0.101
	M	**0.854**	**0.927**	**0.791**	**0.904**	**0.873**	**0.688**	**0.916**	**0.638**	1.029	**0.011**	**0.096**
YOLOv9	R	0.802	0.879	0.737	0.895	0.812	0.609	0.982	0.685	1.108	0.014	0.105
	M	**0.828**	**0.9**	**0.767**	**0.901**	**0.85**	**0.655**	0.974	0.685	1.19	**0.012**	**0.099**
YOLOv10	R	**0.869**	**0.93**	**0.815**	**0.945**	**0.887**	**0.762**	1.553	0.967	1.82	**0.004**	**0.056**
	M	**0.882**	**0.926**	**0.842**	**0.946**	**0.906**	**0.751**	**0.924**	**0.528**	1.606	**0.003**	**0.054**
A.	R	*0.826*	*0.897*	*0.766*	*0.913*	*0.826*	*0.618*	*0.903*	*0.602*	*0.994*	*0.011*	*0.088*
	M	*0.827*	*0.895*	*0.769*	*0.912*	*0.836*	*0.612*	*0.774*	*0.518*	*0.993*	*0.010*	*0.087*
	*A*	*0.827*	*0.896*	*0.767*	*0.913*	*0.831*	*0.615*	*0.839*	*0.560*	*0.994*	*0.010*	*0.088*

*Notes* Data Type R-RGB, M-Multispectral, vx v-Version and x- range 5–10, A. Average, Prec Precision, mAP mean Average Precision, All experiments were conducted using the small (S) variants of each architecture.

Rec Recall, Acc Accuracy, MSE Mean Squared Error, MAE Mean Absolute Error, DFL -Distribution Focal Loss.

**Fig 7:**
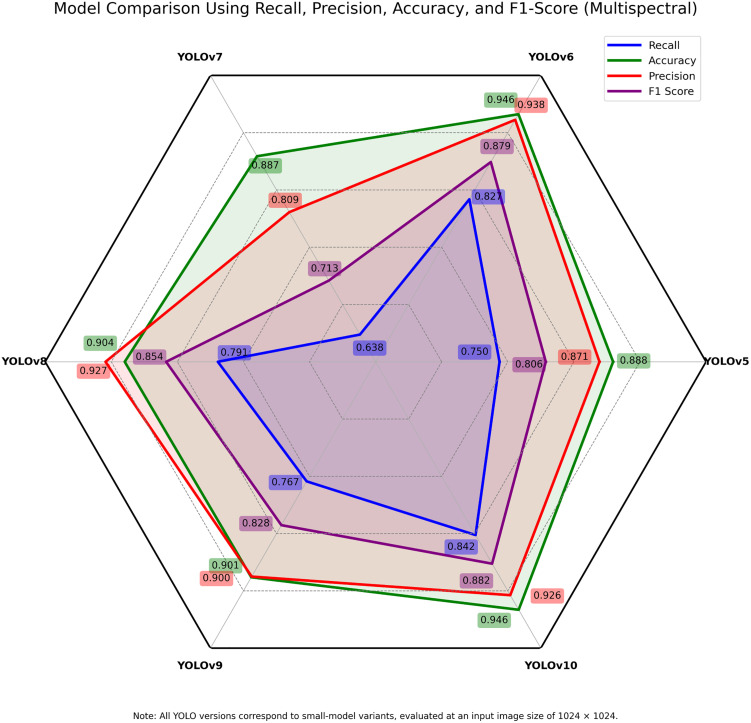
Performance metrics on the multispectral (OCN) dataset across evaluated YOLO models. The figure presents accuracy, precision, recall, and F1-score values obtained using multispectral imagery.

**Fig 8:**
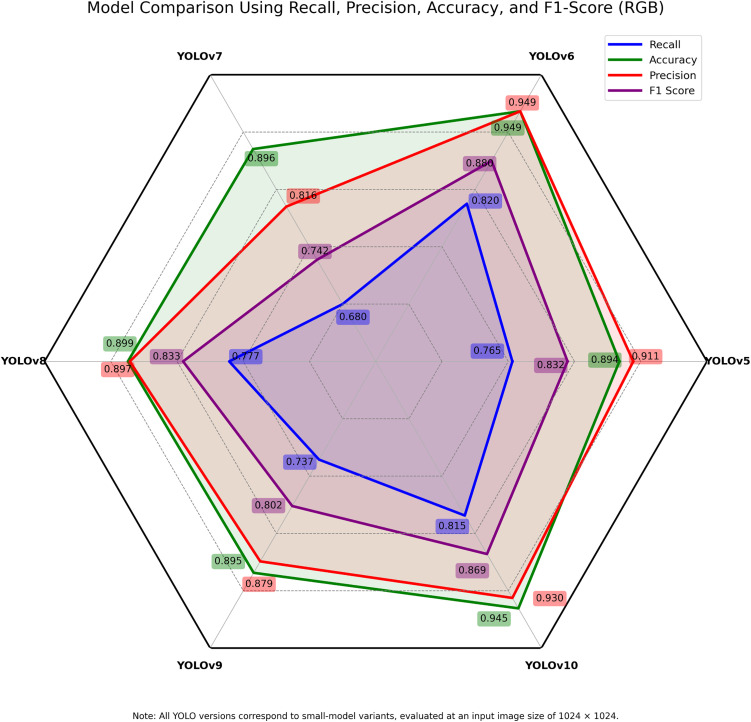
Performance metrics on the RGB dataset across evaluated YOLO models. The figure presents accuracy, precision, recall, and F1-score values obtained using RGB imagery.

The accuracy, precision, recall and F1-score representations of the multispectral and RGB datasets are presented in **Figs 7** and **8** respectively. YOLOv5 demonstrated good results, particularly in the RGB precision (0.911) and accuracy (0.894) (**Table 4**) with a marginally lower result compared with the multispectral dataset. It was a good foundation, despite its older version. It, however, had constraints in recall and F1-score, which is the inability to recognize latent features of disease. YOLOv6 also exhibited a significant positive performance relative to its input metrics with both RGB and multispectral inputs showing good balance. The depth-wise convolutions and better bottleneck structures of it also helped in its good generalisation, especially in its F1-score and its accuracy.

YOLOv7 was performing poorly on both types of data with the lowest recall and F1-scores. It had a high dependence on anchor-based detection, which probably was an impediment of its efficiency under intricate lighting and texture circumstances. A superior balance in precision, in terms of recall, and F1-scores was achieved in YOLOv8 through an enhanced Path Aggregation Network (PAN) and CSPDarknet backbones. The fact that it is very reliable implies it can be deployed in the field with limited computing power.

YOLOv9, which features enhanced spatial attention and convolution, showed slight improvements in F1-score and recall with multispectral data, compared with the model averages. This means that the disease patterns having low RGB contrast are handled better. YOLOv10 was the most successful of all models. The multispectral data recorded higher superior precision (0.926), recall (0.842), and accuracy (0.946) (**Table 4**) with high F1-scores in both data types. The improved anchor-free modules and transformer-based attention mechanisms enabled enhanced localisation and classification accuracy. These architectural features maintained robust performance even under varying leaf textures. The small difference between the RGB and multispectral scores proved that it is reliable with various modalities. In the majority of models, multispectral data performed better. It is able to access near-infrared data hence it is more advantageous in segmenting diseases and early detection. This was most noticeable in YOLOv5, YOLOv7 and YOLOv9, where RGB imaging struggled under natural lighting conditions.

Overall, spectral diversity coupled with the latest object detection systems have demonstrated the ability of having a significant increase in disease recognition potential. YOLOv10 and YOLOv8 have the best current structure due to their architectural enhancements and data strength that makes it the most up-to-date model in early-stage mango leaf disease detection. Meanwhile, YOLOv6 can also be highlighted as a highly efficient option when it comes to real-time implementation on edge devices as a result of its balanced metrics and optimised architecture.

#### Assessment and analysis of mAP@50 and mAP@50–95 of YOLO models.

**Fig 9:**
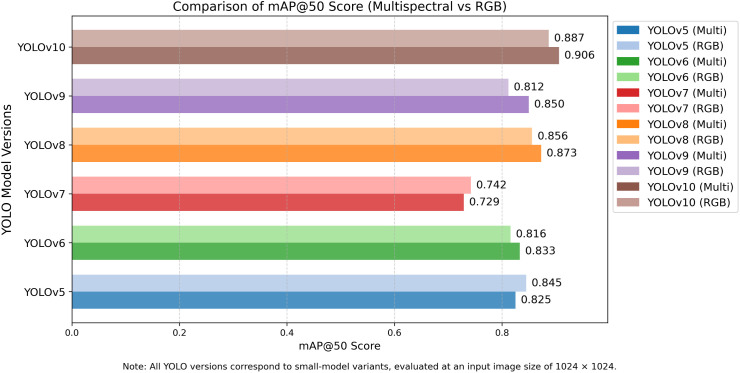
Comparison of mAP@50 scores for YOLOv5-YOLOv10 models evaluated on RGB and multispectral datasets. The comparison is conducted using the same training and evaluation settings for both RGB and multispectral inputs.

**Fig 10:**
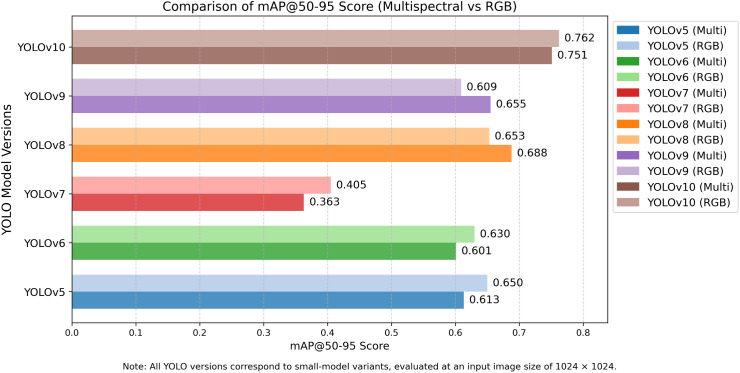
Comparison of mAP@50-95 scores for YOLOv5-YOLOv10 models evaluated on RGB and multispectral datasets. The comparison is conducted using the same training and evaluation settings for both RGB and multispectral inputs.

The comparison of YOLO models on mAP@50 and mAP@50–95 is an evaluation that provides end-to-end object detection performance on localising and classifying mango leaf diseases due to various Intersection over Union (IoU) levels. These indicators provide the understanding of the gross and specific detection abilities, particularly of real-time agricultural monitoring with the use of RGB and multispectral UAV images. The comparison of the YOLO model performance in terms of mAP@50 and mAP@50–95 for both datasets is illustrated in **Figs 9** and **10**. YOLOv5 is reliable, with mAP@50 (multispectral) and mAP@50 (RGB) of 0.845 and 0.825 (**Table 4**) respectively, which is better than the average baselines of 0.836 and 0.826. It also boosts its performance by using adaptive anchor boxes and feature fusion that is more useful during multispectral processing. It is however less accurate when dealing with complicated aspects of disease such as dieback and sooty mould and is hence not the most useful in fine grain disease localisation.

YOLOv6 achieved a mAP@50 of 0.833 on RGB, and 0.816 (**Table 4**) on multispectral images, suggesting stronger performance on RGB inputs. This is because its feature extraction pipeline is optimised for visible-spectrum imagery It has good performances with the diseases that have a definite surface texture like bacterial canker. It is a good detector, but segmentation is mediocre with mAP@50–95 scores of 0.601-RGB, 0.630-multispectral, and its advantage is in multispectral-enhanced lesion segmentation.

Unlike YOLOv6, YOLOv7 recorded the lowest mAP@50 values of 0.742 (multispectral) and 0.729 (RGB) (**Table 4**), indicating challenges with small or faint lesions such as anthracnose. The marginal difference between RGB and multispectral scores indicates that spectral depth is not well utilised, likely due to the absence of attention mechanisms. Correspondingly, mAP@50–95 score of 0.405 (multispectral) and 0.363 (RGB) (**Table 4**) reflects poor bounding box accuracy. YOLOv8 also performs well with mAP@50 of 0.873 (RGB) and 0.856 (multispectral). Its anchor-free detection and augmented feature pyramid also offer strong detection in RGB inputs (0.688), and in multispectral (0.653) inputs also support the fact that it is generalised across both data modalities.

YOLOv9 has 0.850 (RGB) and 0.812(multispectral) mAP@50 (**Table 4**) with the support of CSPNet and SPP layers. Its reduced performance marginally in multispectral performance implies inefficiencies in the early spectral fusion. At mAP@50–95 of 0.655 (RGB) and 0.609 (multispectral), the YOLOv9 is good at general localisation but not at soft-edged lesions such as sooty mould. Due to the attention modules and high convolutional design, YOLOv10 is ranked first in mAP@50: 0.906 (RGB) and 0.887 (multispectral). It is effective in high-level and local disease characteristics, being superior at the real-world detecting of aberrant symptoms. YOLOv10 has mAP@50–95 scores of 0.762 (RGB) and 0.751 (multispectral), which indicate the higher accuracy and stability of the bounding boxes of different IoU thresholds. The small disparity in its performance highlights its flexibility in both the RGB and multispectral systems.

Mean values of mAP@50 of models are 0.612 (multispectral) and 0.618 (RGB) (**Table 4**). Although it is slightly surpassed by RGB in fine-grained localisation, multispectral is essential in improving contrast in the detection at the early growth stages, which upholds its usefulness in precision agriculture applications. The general performance of the YOLOv10 was the highest in both metrics and type of input, then came YOLOv8 and YOLOv6. The findings reveal the importance of the architecture additions such as attention modules and spectral-aware design to detect the agricultural diseases accurately.

### Computational efficiency evaluation

When implementing deep learning models in real-time agricultural monitoring via UAV platforms, the high detection accuracy is not the only requirement; edge device-computational efficiency is also required. The NVIDIA Jetson Orin Nano and Raspberry Pi 5are widely used in aerial phenotyping and disease detection under strict resource and power constraints. Evaluating models by architectural depth (layers), parameters count (millions), computational cost (GFLOPs), and inference time (milliseconds(ms)/seconds(s)) are therefore essential. Such metrics determine responsiveness of the model, power consumption, and ability to deploy in a dynamic farm set-up. Detailed comparison given in YOLOv8 vs YOLOv10 Trade-off section This discussion aids in the choice of models in precision agriculture where aerial monitoring with high-frequency and the latency-sensitivity is of concern.

### Confusion matrix data

#### Multispectral comparative evaluation.

**Fig 11:**
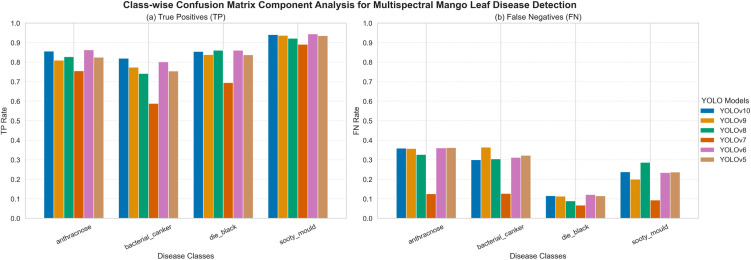
Confusion matrix components illustrating class-wise TP and TN prediction performance of YOLOv5-YOLOv10 models on the multispectral dataset. The analysis covers four mango leaf disease classes: anthracnose, bacterial canker, dieback, and sooty mould.

**Fig 11** shows the TP (True Positive) and FN (False Negative) of both models using the UAV-based multispectral imagery regarding class-wise analysis of the YOLOv5-YOLOv10 model. YOLOv8 and YOLOv10 will give a better TP value on most disease classes that include anthracnose and sooty mould, which illustrates a better evidence of detection. In this case, YOLOv5 has larger FN values, particularly anthracnose, implying that they are less sensitive to fine disease characteristics. Bacterial canker also has rather high FN rates among models since it has a spectral similarity with the surrounding leaf textures. On the whole, YOLOv8 and YOLOv10 lower the FN rates and show a superior ability to detect multispectral diseases.

#### Comparative analysis based on RGB data.

**Fig 12:**
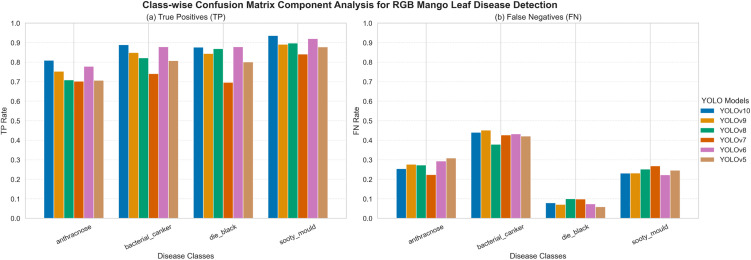
Confusion matrix components illustrating class-wise TP and TN prediction performance of YOLOv5-YOLOv10 models on the RGB dataset.

**Fig 12** is the results of the TP and FN per class of the YOLOv5-YOLOv10 model on RGB images. The bacterial canker and sooty mould have higher TP values in most of the models, which means that such diseases have more vivid visual patterns in RGB images. Conversely, anthracnose and dieback exhibit lower TP and the higher values of FN because they have subtle symptoms. The previous models like YOLOv5 and YOLOv6 have a higher FN, especially in the case of anthracnose and bacterial canker which is worse sensitivity to complex or low contrast disease patterns. Comparatively, YOLOv8 and YOLOv10 decrease FN rates, which indicate the better feature extraction and detection ability. Nevertheless, the detection based on RGB remains relatively high in terms of FN values in comparison to the multispectral outcomes, which points to the benefit of using extra spectral data to discriminate diseases.

#### Normalised confusion matrix insights.

**Fig 13:**
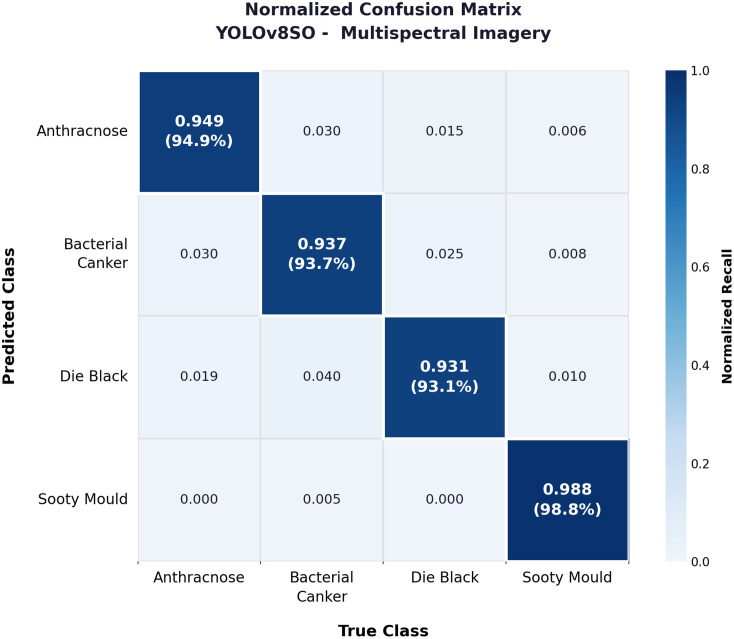
Normalized confusion matrix for the YOLOv8SO model evaluated on the multispectral dataset. The matrix highlights highlighting class-wise prediction accuracy across the evaluated disease categories.

**Fig 13** is the normalized confusion matrix of YOLOv8SO on the multispectral dataset taken as a result of a single inference run. The four classes did well on the diagonal – sooty mould topped the charts with a score of 0.988, then came anthracnose with a score of 0.949, bacterial canker −0.937 and dieback −0.931. It is not surprising that the result is the strong sooty mould. Its whitish powdered surface is distinctly different in terms of reflectance profile as compared to the other three diseases with multispectral bands, leaving the model with very little margin to doubt when making a prediction.

Dieback exhibited inter-class confusion highest with 4% of its cases forecasted as bacterial canker. In early stage both diseases may appear similar, with blackened necrotic tissue on stems exhibiting surface textures resembling canker lesions. This has been found observed in both field inspections and model predictions. There is a less significant two-way misidentification between anthracnose and bacterial canker (3% each) that is based on the same reasoning given that they both appear as dark discolouration on both leaf and stem surfaces. However, none of the off-diagonal values exceeded 0.05, and this preserves the integrity of overall classification. Combined, the matrix demonstrates that multispectral imaging measures sufficient spectral resolution among types of diseases so that the model can make robust distinctions, even between visually similar ones.

### Spectral responses evaluations

#### Multispectral Vs RGB comparison.

In this study, results are analysed using both multispectral and RGB images. As much as the surface appearance is recorded by the RGB images, multispectral imaging records a slight physiological alteration of a disease at early stages. **Table 4** demonstrates that YOLOv10 and YOLOv8 were better at working with multispectral data (F1 - 0.882, mAP@50–0.906) than with RGB (F1 - 0.869, mAP@50–0.887). In all YOLO models, multispectral data always enhanced recall and reduced false negatives, especially with anthracnose and dieback, leading to a slight increase in average F1 (0.827 vs. 0.826), precision (0.895 vs. 0.897), and accuracy (0.912 vs. 0.913). All in all, multispectral models showed the best performance in terms of UAV-based disease detection in all versions tested. A mean value of all models’ metrics is used as a benchmark in the future comparison to attain an effective model as presented in **Table 4**. Additional details on model complexity, inference efficiency, including the number of layers and parameters, GFLOPs, and inference latency for YOLOv5-YOLOv10 across RGB and multispectral datasets, are provided in supplementary material (S1 Table).

#### Comparison of spectral band response.

Anthracnose is a disease that intervenes with the cellular level and kills chlorophyll resulting in the loss of green colour, thus the affected areas will reflect lower in the NIR compared to the healthy tissue. The lesions are sensitive to NIR and cyan bands. The deep tissue damage leaves a high spectral imprint that improves the efficiency of OCN. An oily margin and a chlorotic margin are the result of the bacterial canker. The bacteria do not destroy most of the internal structure of the leaf; consequently, spectral variation is not so distant to the healthier leaves. Such a bright colour difference is easily represented in RGB pictures (**Fig 17**), but OCN (**Fig 18**) does not give much of a choice.

Dieback leads to the loss of foliage and discoloured vascular tissues are exhibited signifying heavy loss of water and chlorophyll breakdown and a sharp decrease in NIR reflectance. Also, cyan band responds to early pigment changes. The cellular collapse is accentuated by the multispectral imaging before leaves turn completely shrivelled but the RGB only shows the browning (**Fig 17**). Sooty mould produces a velvety black layer on leaf surface, it does not infect cells of leaves, blocks light and inhibits photosynthesis, covering the chloroplasts. This significantly reduces the visible and NIR reflectance (**Fig 18**). Nevertheless, the severe NIR difference between the normal and affected provides an advantage.

### Optimal YOLO model ranking of individual class of disease

As seen in **Tables 5** and **6**, there was a comparative analysis done between YOLOv5 and YOLOv10 architectures with the same datasets and with the same training parameters. The models were tested on two modalities of images namely RGB and multispectral, and each mode was annotated class-wise. Measures of evaluation were precision, recall, F1-score and average precision at 0.5 IoU (mAP@50). From **Tables 5** and **6**, values exceeding the average (“A”), as reported in the row labelled “A”, are highlighted in bold. The average values in the “A” row serve as the baseline for comparison, against which the performance of the benchmarked algorithms is evaluated. Based on this baseline analysis, the most effective model is subsequently identified and selected.

**Table 5:** Class wise performance comparison data for anthracnose and bacterial canker.

YOLO Version	Data Type	Class Data									
		Anthracnose					Bacterial canker				
		P	R	F1	mAP@50	mAP@50–95	P	R	F1	mAP@50	mAP@50–95
YOLOv10	M	**0.926**	**0.832**	**0.880**	**0.904**	**0.738**	**0.928**	**0.794**	**0.856**	**0.862**	**0.696**
	R	**0.932**	**0.727**	**0.817**	**0.843**	**0.702**	**0.931**	**0.832**	**0.879**	**0.895**	**0.762**
YOLOv9	M	*0.908*	0.730	0.810	**0.843**	**0.635**	*0.897*	*0.673*	*0.769*	*0.768*	*0.571*
	R	0.837	0.609	0.705	0.705	0.488	0.881	0.745	0.807	0.815	0.610
YOLOv**8SO**	M	**0.988**	**0.949**	**0.968**	**0.974**	**0.93**	**0.993**	**0.937**	**0.964**	**0.968**	**0.911**
YOLOv8	M	**0.934**	**0.764**	**0.840**	**0.872**	**0.677**	**0.925**	**0.705**	**0.800**	**0.810**	**0.615**
	R	**0.882**	0.658	**0.754**	**0.761**	**0.537**	0.883	**0.787**	**0.832**	**0.862**	**0.657**
YOLOv7	M	0.844	0.666	0.740	0.763	0.384	0.801	0.459	0.584	0.592	0.240
	R	0.819	0.620	0.706	0.704	0.347	0.827	0.675	0.743	0.738	0.368
YOLOv6	M	**0.937**	**0.816**	**0.870**	*0.822*	0.589	**0.927**	**0.725**	**0.814**	0.733	0.464
	R	**0.939**	**0.701**	**0.803**	0.698	0.514	**0.957**	**0.819**	**0.883**	0.814	0.607
YOLOv5	M	0.907	0.724	0.810	0.829	*0.628*	*0.900*	*0.677*	0.773	**0.771**	**0.572**
	R	0.841	0.643	0.729	0.724	0.506	0.848	0.773	0.809	**0.830**	0.616
A.	M	0.909	0.755	0.825	0.839	0.609	0.896	0.672	0.766	0.756	0.526
	R	0.875	0.660	0.752	0.739	0.516	0.888	0.772	0.825	0.826	0.603

P-Precision, R- Recall, F1-F1 score, mAP -mean Average Precision at IoU 0.5 (mAP@50).and 0.5 to 0.95 (mAP@50–95). YOLOv8SO -YOLO v8 SeqOpt, A-Average. All experiments were conducted using the small (S) variants of each architecture.

**Table 6:** Class wise performance comparison data for dieback and sooty mould.

YOLO Version	Data Type	Class Data									
		Dieback					Sooty mould				
		P	R	F1	mAP@ 50	mAP @50–95	P	R	F1	mAP @50	mAP@ 50–95
YOLOv10	M	**0.91**	**0.816**	**0.86**	**0.892**	**0.718**	**0.939**	**0.925**	**0.932**	**0.966**	**0.852**
	R	**0.913**	**0.798**	**0.852**	**0.871**	**0.713**	**0.943**	**0.902**	**0.922**	**0.939**	**0.872**
YOLOv9	M	0.876	0.778	0.824	0.844	0.636	0.918	0.888	0.903	**0.946**	**0.779**
	R	0.882	0.768	0.821	0.839	0.589	0.914	0.827	0.868	0.89	0.749
YOLOv**8SO**	M	**0.984**	**0.931**	**0.957**	**0.964**	**0.924**	**0.996**	**0.988**	**0.992**	**0.993**	**0.97**
YOLOv8	M	**0.912**	**0.798**	**0.851**	**0.858**	**0.66**	**0.937**	**0.896**	**0.916**	**0.953**	**0.801**
	R	**0.896**	**0.799**	**0.845**	**0.876**	**0.63**	**0.929**	**0.864**	**0.895**	**0.925**	**0.79**
YOLOv7	M	0.715	0.597	0.651	0.663	0.278	0.878	0.83	0.853	0.898	0.549
	R	0.762	0.641	0.696	0.672	0.347	0.857	0.785	0.819	0.854	0.557
YOLOv6	M	**0.943**	**0.851**	**0.895**	**0.857**	**0.63**	**0.945**	**0.918**	**0.931**	0.922	0.719
	R	**0.934**	**0.867**	**0.899**	**0.86**	**0.661**	**0.965**	**0.894**	**0.928**	**0.892**	0.74
YOLOv5	M	0.904	0.767	0.83	0.837	0.623	0.932	0.891	0.911	0.945	0.776
	R	0.884	0.741	0.806	0.844	0.574	0.911	0.841	0.875	0.9	0.756
A.	M	0.877	0.768	0.818	0.825	0.591	0.925	0.891	0.908	0.938	0.746
	R	0.879	0.769	0.82	0.827	0.586	0.92	0.852	0.885	0.9	0.744

P-Precision, R- Recall, F1-F1 score, mAP -mean Average Precision at IoU 0.5 (mAP@50).and 0.5 to 0.95 (mAP@50–95). YOLOv8SO -YOLO v8 SeqOpt, A-Average. All experiments were conducted using the small (S) variants of each architecture.

YOLOv10 was the best among them with 0.946 mAP@50 and 0.911 F1-score (**Table 4**) on the multispectral dataset due to its hybrid transformer-CNN architecture and its sophisticated attention mechanisms. Nevertheless, it had high inference latency and had large model size, which limited its deployment. YOLOv8 was the most balanced with an mAP@50–0.903 (**Table 4**), F1- score of 0.896 and inference efficiency, especially on small and clustered lesions, which suggests its inference time capabilities in real-time. YOLOv8 was therefore put on the shortlist to be optimised and deployed further. Multispectral data was always superior to RGB equivalents in all versions of YOLO, which once again confirms the role of spectral diversity in the early symptoms of diseases. By way of example, YOLOv6 outperforms multispectral data with an mAP@50–0.873 vs. RGB with 0.823, indicating the usefulness of the NIR and Cyan bands in addition to the RGB bands used to emphasize chlorosis and necrotic symptoms. YOLOv5 showed very good results on RGB imagery (F1-score = 0.852, mAP@50 = 0.833), which indicates that it remains useful in situations where only a small amount of spectral information is needed.

### Model selection based on the proposed custom score

Besides the traditional benchmarking measures, a weighted scoring system was adopted to assist in the model choice to solve the real-time mango leaf disease detection using the UAV. This score should be used to facilitate deployment-oriented comparison with operating conditions that are real-time and not to maximize post-facto accuracy. Here, mAP@50 and F1-score (**Table 4**) were highlighted because they focus on different facets of localization accuracy and classification reliability that would be used and important in making decisions in the field in the moment. The weighted score (1) that has been utilized in the paper is calculated as:


$$\begin{array}{c} \text{Score}=(\text{F1 x0}.\text{4})+(\text{mAP}@\text{50 x 0}.\text{6}) \end{array}$$ (1)

The F1-score was weighted (0.4) to represent the part it plays in the process of balancing precision and recall in order to confirm immediately the presence of a disease. A higher weight of 0.6 was assigned to mAP@50 as precise spatial localization of disease-affected areas is essential for real-time UAV inspection, prioritising accurate bounding box detection over offline post-processing refinement. The most challenging mAP@50–95 measure, although included for comprehensive benchmarking, was not given priority in the weighted score, as it evaluates performance across multiple mAP thresholds that are not as critical under real-time latency and resource constraints. Alternative weight combinations (e.g., equal or more mAP-dominant weightings) were examined during preliminary analysis, and they did not alter the relative ranking of the models.

**Fig 14:**
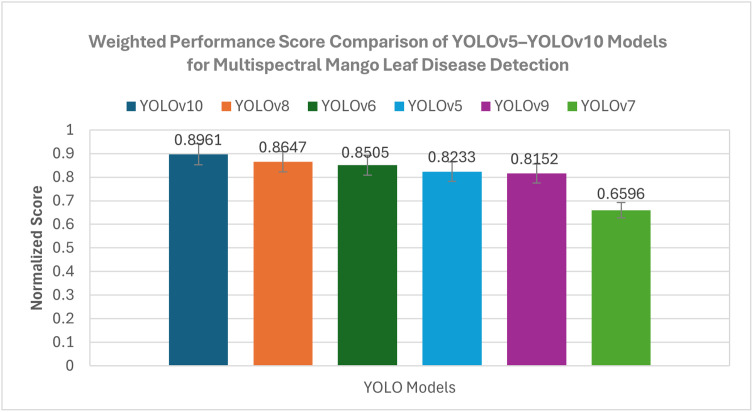
Comparison of custom weighted performance scores for YOLOv5-YOLOv10 models on the multispectral mango leaf disease dataset. The weighted score integrates multiple performance and efficiency metrics to provide an overall comparative assessment of model effectiveness.

The weighted score (Equation 1) was calculated separately in each category of diseases (anthracnose, bacterial canker, sooty mould and dieback) and the average score of all the categories compared. **Fig 14** shows the comparison of the weighted score (Equation 1) of YOLOv5 through YOLOv10. YOLOv10 was the best weighted (0.896), which is mainly because it has been doing very well in terms of mAP@50 and F1-score across the disease classes. YOLOv8 was next with a weighted mark of 0.865, which has a relatively lower detection accuracy but more preferable real-time performance features. YOLOv6 scored 0.851, which is a weighted score, meaning that it performs competitive generalization.

Although YOLOv10 had a better performance with respect to the raw detection measures compared to the other models, its increased computational cost reduced its application in real-time in edge deployment. Based on these findings, YOLOv8 was chosen as the balanced model architecture, offering a favourable trade-off between detection accuracy, inference latency, and memory consumption for deployment on embedded platform, namely Jetson Orin Nano and Raspberry Pi 5.

### YOLOv8 vs YOLOv10 trade-off

In addition to the detection accuracy (as mentioned on Tables 4–6), there are evident differences in complexities and deployment in the comparison of YOLOv8 and YOLOv10. YOLOv10 uses a significantly more complex architecture with 402 layers, as opposed to 225 layers in YOLOv8 (S2 Table), and it results in significantly more representational capacity at the cost of using more memory during training (13.6 GB versus 11.6 GB). Its additional layers introduce extra memory and execution costs on running on edge devices using only low-power and CPU-only systems like the Raspberry Pi 5. YOLOv8 is built on a shallower network architecture, which makes it less memory intensive on deployment.

The loss behaviour in training and validation also depends on the difference in the architectural complexity. On multispectral training using SGD the YOLOv10 has higher loss values, with training box, classification, and Distribution Focal (DFL) loss of 1.30, 0.78, and 1.70, respectively, and validation loss of 0.92 (box), 0.53 (classification), and 1.61 (DFL) (**Table 4**). Compared to it, YOLOv8 has lower training losses of 0.40 (box), 0.28 (classification), and 0.82(DFL) and lower validation losses of 0.92 (box), 0.64 (classification) and 1.03(DFL). In the comparison of loss components across the assessed conditions, YOLOv10 consistently exhibited higher loss values than of YOLOv8. Therefore, YOLOv8 was selected for edge deployment assessments.

### Impact of optimiser blending on YOLOv8

When the SeqOpt optimiser, which combines sequential execution of SGD and AdamW, was integrated into the YOLOv8 training pipeline, it yielded a significant improvement in model performance for crop disease detection. The SeqOpt method outperformed the standard SGD training with the following results: it increased precision (0.927(SGD)) to (0.984(SeqOpt)), recall (0.791) to (0.953), and the F1-score (0.854) to (0.968) (**Table 7**). Also, the average precision at an IoU threshold of 0.5 (mAP@50) improved to 0.984 as opposed to 0.873, and mAP@50–95 increased to 0.914 as compared to 0.688.

**Table 7:** Optimiser ablation and top candidate comparison on multispectral data.

S. No.	Model	Data type	Optimiser 1	Optimiser 2	Precision	Recall	F1-score	mAP@50	mAP@50–95
1	YOLO v10*	Multispectral	SGD	–	0.926	0.842	0.882	0.906	0.751
2	YOLOv8		SGD	–	0.927	0.791	0.854	0.873	0.688
3			AdamW	–	0.932	0.901	0.911	0.927	0.784
4			SGD	AdamW	**0.984**	**0.953**	**0.968**	**0.984**	**0.914**

*YOLOv10s to indicate it is a reference baseline, not part of the ablation. The YOLOv8 with SGD and AdamW optimiser indicates the YOLOv8SO.

These notable improvements indicate that the progressive optimisation strategy developed by SeqOpt successfully models non-random disease symptoms such as clustered and overlapping lesions ones capturing disease manifestations even when observed in different lighting and background conditions. This optimisation platform expedites convergence and improves the robustness and generalisation of YOLOv8 on multispectral agricultural data, and its application has the potential to assist in the advancement of real-time monitoring of crop diseases.

### Ablation analysis

The **Table 7** demonstrates the optimiser ablation of YOLOv8 on multispectral data (averaged score), which includes YOLOv10 as a reference baseline. In all conditions, SeqOpt was better than SGD-only and AdamW-only training, achieving the highest F1-score and mAP@50, and has lower computational overhead, which explains why it was chosen as the final deployment model.

### Real-time edge deployment in embedded platforms

The deployment test was aimed at determining the practicality of the optimised YOLOv8 model in real-time using both PyTorch (.pt) and ONNX formats on a Raspberry Pi 5 and NVIDIA Jetson Orin Nano edge devices. All models were evaluated using pre-captured RGB and multispectral OCN images resized to 1024 × 1024 pixels, and identical datasets, data splits, and evaluation protocols were applied across all experiments. Each inference procedure was implemented through scripted batch inference, without GPU acceleration, to provide realism to the low-cost field applications. To allow a granular analysis, the overall execution time per image was broken down into preprocessing, inference, and postprocessing latencies.

**Fig 15:**
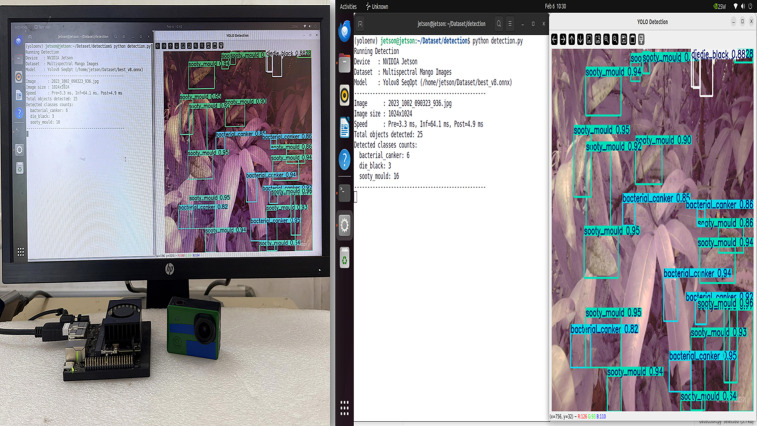
Hardware deployment and testing setup for the SeqOpt-trained model on Single-Board Computer (SBC) – Jetson. The figure shows the NVIDIA Jetson Orin Nano (Left) and Screenshot of predicted output generated during deployment (right side).

**Fig 16:**
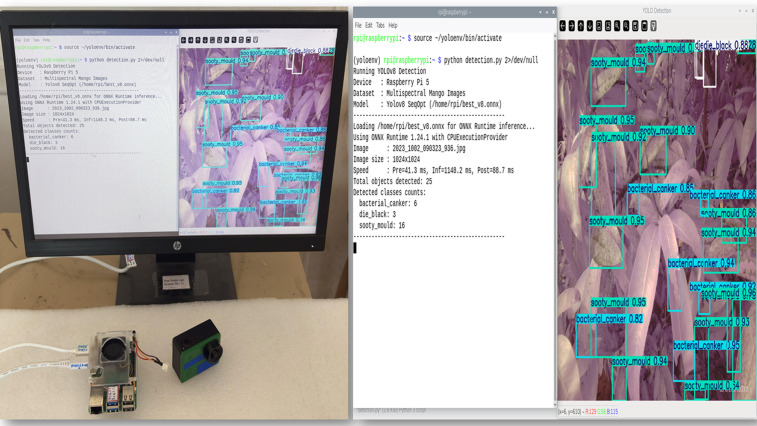
Hardware deployment and testing setup for the SeqOpt-trained model on SBC - RPi-5. *The figure* show*s* the Raspberry Pi 5 (Left) and Screenshot of predicted output generated during deployment (right side).

Raspberry Pi 5 (**Fig 16**) and the NVIDIA Jetson Orin Nano (**Fig 15**) were used in deployment experiments in order to test the behaviour of the model on different constraints linked to edge computing. Inference was performed using a CPU-only execution on the Raspberry Pi 5. In these resource-constrained configurations, the per-image runtimes of all the models considered were of the order of seconds. The tested architectures also showed that the YOLOv8 model optimised with the suggested Sequential SGD-AdamW (SeqOpt) strategy showed the most reasonable trade-off between the detection accuracy and efficiency on multispectral data. All values are reported as mean measurements from three inference runs conducted on the edge device. For this configuration, (YOLOv8SO) the F1-score reached 0.968 and the mAP@50 reached 0.974 (S3 Table. - RPi5 ONNX deployment results), while the corresponding per-image runtime and energy consumption were about 1.20 s and 0.000445 Wh (Watt-hour) per image. Comparatively, the default YOLOv8 multispectral model based on SGD-only took a mean of 1.61 s per image and used 0.000592 Wh per image, with a lower detection accuracy. This result represents a reduction of 25.2% in inference latency and 24.8% in energy consumption relative to the SGD-only baseline. It is noted that YOLOv6 and YOLOv7 were not evaluated under ONNX testing, because the needed conversion libraries and toolchain support was not available in such architectures. To make the figures clearer when several models and image modalities are provided simultaneously, the model’s name and the data types are concatenated in the legend labels – i.e. YOLOv8SOMulti is the SeqOpt-trained YOLOv8 model which was evaluated on multispectral data. It is consistent with the supporting tables.

The performance trends of both PyTorch (.pt) and ONNX deployments were similar on the Raspberry Pi 5. This implies that in CPU-only execution, the run time is normally limited by hardware capability and less by the model format or engine. Due to this, there was a similar gain in the relative performance of the SeqOpt optimisation strategy between deployment formats on this platform.

When deployed on an NVIDIA Jetson Orin Nano, inference times were significantly reduced, indicating the application of GPU acceleration, with the per-image runtimes represented in the milliseconds range. To prevent confusion and inconsistent reporting, all the run times are expressed in a common scale in the respective figures. When evaluated using the PyTorch (.pt) format, the SeqOpt-trained YOLOv8 multispectral model recorded a mean inference (pre-processing + inference + post-processing) time of 72.0 ms per image, with an F1-score of 0.97 and an mAP@50 of 0.975 (S4 Table. – NVIDIA Jetson PyTorch deployment results). The corresponding average energy consumption was 0.000281 Wh per image. The default YOLOv8 model trained only with SGD had a runtime value of 76.6 ms per image and inferior performance in detection. Similar performance trends were observed in the ONNX format which means that the noticed enhancement should be rather explained by the optimising strategy than the format of deployment.

Overall, these findings suggest that hardware capability is the primary factor of absolute runtime performance, though the SeqOpt optimisation strategy provides a better accuracy-efficiency trade-off between heterogeneous edge devices. Results of Raspberry Pi 5 show that it can operate in a very strict set of resource requirements, and the Jetson Orin Nano results show that it can run nearly in real-time with the aid of a set of GPU accelerators.

It is noted that the differences noted between deploying PyTorch (**Figs 19** and **21**) and ONNX (**Figs 20** and **22**) on edge platforms can be mainly explained by the hardware-specific runtime optimisation instead of architecture-level alterations. Optimised CPU execution paths can be useful with ONNX inference, but PyTorch is much more directly expressible in terms of tightly integrated CUDA (Compute Unified Device Architecture) pipelines when using a platform with a graphics card such as the NVIDIA Jetson Orin Nano. These effects were not considered to be paramount in the context of the current work, and the dynamics of the relative performance of model configurations were stable in the deployment types.

**Fig 17:**
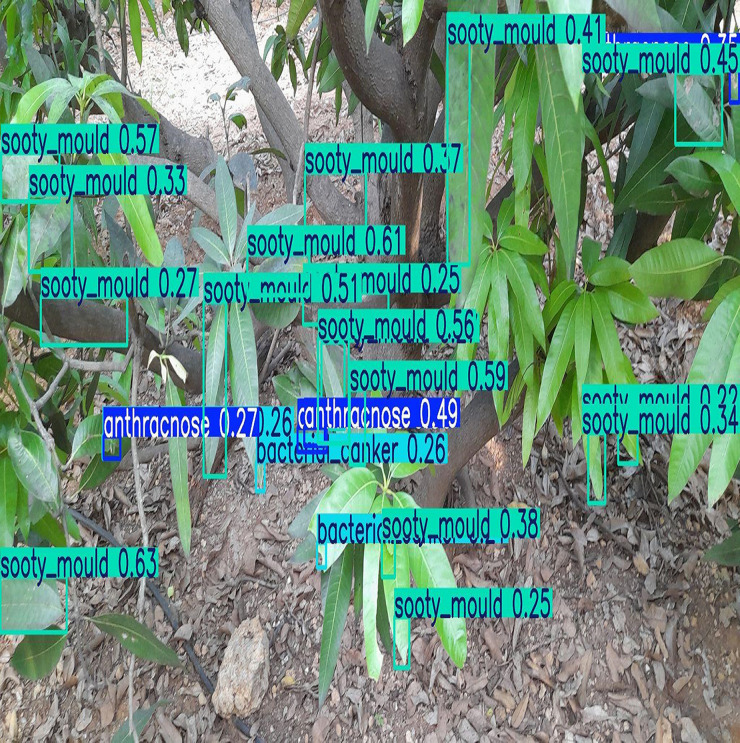
Qualitative detection results on the RGB dataset. This figure demonstrates bounding box localization and classification of mango leaf disease instances**.**

**Fig 18:**
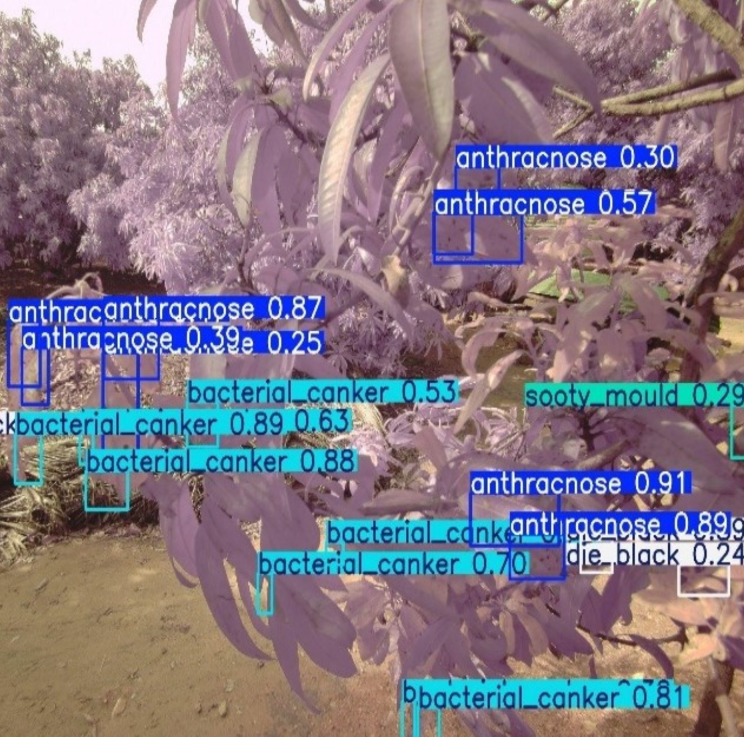
Qualitative detection results on the multispectral dataset. This figure illustrating disease localization under varying illumination conditions.

The deployment experiments on NVIDIA Jetson Orin Nano and Raspberry Pi 5 are illustrated in **Fig 15** and **Fig 16** respectively. The qualitative predictions of the RGB and multispectral datasets (**Figs 17** and **18**, respectively) demonstrate accurate localization of the bounding box and satisfactory detection of small, clustered, or partially occluded lesions. In **Fig 18**, the multispectral results demonstrate that the lesions can be easily defined, even with a certain portion of non-uniform illumination used to obtain multispectral images.

**Fig 19:**
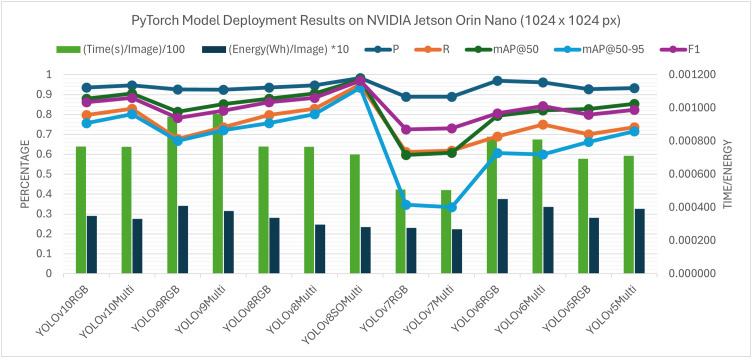
Validation results of the YOLOv5 to YOLOv10 multispectral model deployed on the NVIDIA Jetson Orin Nano using the PyTorch (.pt) format. Results correspond to inference on 1024 × 1024 input images, with per-image latency in the second range and GPU-accelerated execution.

**Fig 20:**
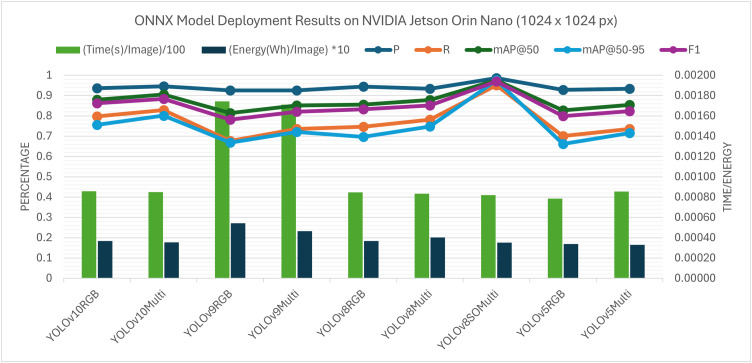
Validation results of the YOLOv5 to YOLOv10 multispectral model deployed on the NVIDIA Jetson Orin Nano using the ONNX format. Inference was performed on 1024 × 1024 images, illustrating the effect of model format on latency and detection performance under GPU acceleration.

**Fig 21:**
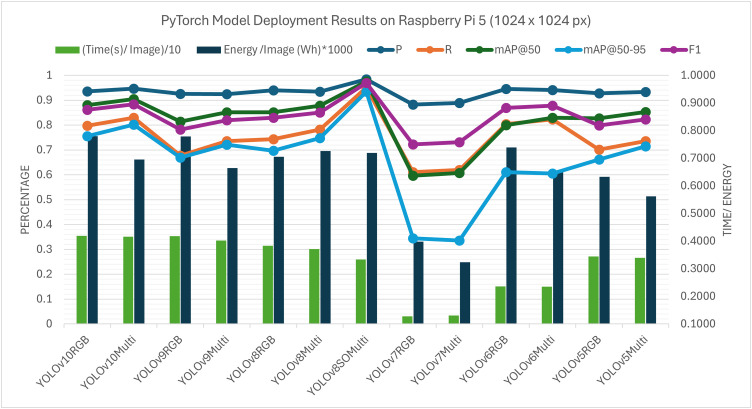
Validation results of the YOLOv5 to YOLOv10 multispectral model deployed on the Raspberry Pi 5 using the PyTorch (.pt) format. Inference was conducted on 1024 × 1024 images using CPU-only execution, with per-image runtime reported in seconds.

**Fig 22:**
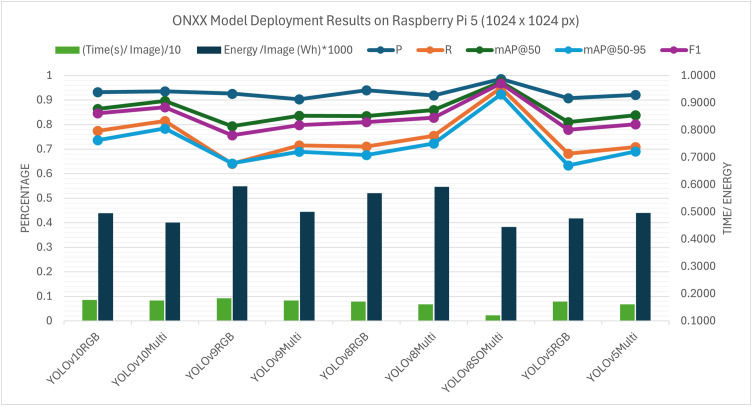
Validation results of the YOLOv5 to YOLOv10 multispectral model deployed on the Raspberry Pi 5 using the ONNX format. Results highlight runtime and detection behaviour for 1024 × 1024 inputs under resource-constrained, CPU-only edge conditions**.**

The trends in the plots in **Figs 19**–**22** indicated that SeqOpt-trained YOLOv8 model maintains favourable runtime and detection performance under embedded deployment conditions. Detailed deployment results are provided in the supplementary material (S2, S3, S4, S5 Tables). Despite the computational constraints of edge platforms, the model demonstrates consistent accuracy and recall, which are often degraded in time-sensitive, resource-limited settings. This test also demonstrated that the suggested deployment pipeline was an accuracy-to-latency trade-off and an entirely optimised, field-ready solution. These improvements have been confirmed by quantitative statistics or qualitative analysis of visual results and highlight the suitability of the optimised YOLOv8 to the real-time monitoring of mango diseases using UAVs on embedded edge computers. It offers a reasonable trade-off in accuracy, latency and energy usage.

#### Protocol of latency and energy measurement.

The inference latency was recorded as the time for the end-to-end processing of every image, comprising preprocessing, model inference, and postprocessing steps. All the latency results were measured under the same conditions across models, with the same test images, input resolution, batch size, model format and fixed runtime setting. Latency values are averaged values reported by repeated inference runs to get rid of transient variation. Custom Python scripts that interacted with the platform power management controls were used to obtain system-level power data when running the inference.

The energy used per image was calculated by averaging total system energy divided by a constant number of runs of inferences, which was in line with the per-image latency measurements reported. It is an average cost of energy to process an image under steady state operating conditions and is suitable in assessing deployment efficiency in real-time UAV applications. To recapitulate closely the conditions of UAV operations in the real-world, every experiment was done in a headless fashion with wireless communication with no display device, keyboard, or mouse attached. SSH (PuTTY) was used for remote access and monitoring, and the same configuration was always kept during all experiments.

Onboard, software-based power monitoring was used to measure energy consumption on Raspberry Pi 5 and Jetson Orin Nano, and the power consumption was not measured with any external inline power meter. In Raspberry Pi 5, the voltage and current of CPU cores were read in the device power management interface with the command tool “vcgencmd pmic_readadc” whereas system-level statistics were recorded with the Python library “psutil.” In Jetson Orin Nano, the real-time power values were read using the built-in power sensors available via the NVIDIA system utility “tegrastats” and Linux “sysfs” interface which is supported by onboard power monitors. Power sampling was set at a constant rate of 1 Hz when inference was being run. Energy consumption was measured by taking the total power time. This was achieved by calculating the average power over time, specifically within the inference window. The average power was then multiplied by the execution duration of the inference process, i.e., the energy reported is the total power consumed by the device during inference. The assessment of energy using the same procedure onboard was performed on the baseline and suggested models to provide equitable, repeated and compatible assessment of energy between platforms.

The measurements of the power were done under fixed and repeatable operating conditions of each platform, such as the same power mode, the same runtime configuration, and background processes. Platform-specific absolute energy values (e.g., watt-hours per image) are reported to be useful to directly compare deployment efficiency in model variants relative to each other but not to provide a universal hardware efficiency standard. Accordingly, relative reductions in latency and energy consumption are emphasized to highlight comparative performance trends under controlled conditions.

### Summary and interpretation

The results of the study confirm the feasibility of combining UAV-based multispectral imaging and optimised YOLO architectures for large-scale detection of mango leaf disease. Optimiser blending can greatly improve the learning of models, and edge deployment can be used to perform inference in the field in real-time. Although YOLOv10 achieved higher detection accuracy, YOLOv8 provided a more favourable balance between accuracy and computational efficiency, making it better suited for deployment on embedded systems. The overall excellent performance of multispectral imagery in all models is an indication that spectral diversity should be emphasized in the future systems to monitor crops. Further, the deployment analysis proves once again the importance of smart resource selection (model format, hardware choice) as a viable application of AI in agriculture. The stated inference latency and throughput demonstrate that the suggested system can handle images captured by UAV in a reasonable amount of time to be able to inspect orchards on a scale. This enables near real-time of disease screening during UAV flights, thus assisting in making the right decisions in time to monitor crops in the field.

## Conclusion and future scope

The study designed and validated a well-constructed UAV-based deep learning system to detect real-time mango leaf disease using custom-built drone which has the MAPIR Survey 3W camera that provides high-quality images of both RGB and multispectral data. The preparation of a robust and generalisable dataset was done by meticulously preprocessing, annotating and creating a balanced dataset to train the YOLO models. Specific design choices in data acquisition and preprocessing addressed challenges related to illumination variation, class imbalance, and image noise, leading to improved detection performance and enabling on-site, real-time disease monitoring in agricultural environment.

Systematic hyperparameter optimisation (learning rate, resolution, custom augmentations, early stopping control, patience, etc.), and optimiser evaluation (AdamW, SGD, RMSprop, etc.) were conducted to achieve the best performance in across UAV-based field scenarios. A controlled and consistent benchmarking of YOLO architectures from YOLOv5 to YOLOv10 was conducted in terms of detection accuracy, inference time, and the ability to be used in real-time edge deployments based on custom datasets. Across these experiments, multispectral dataset performance was much better in comparison with the RGB dataset, especially in the detection at the earliest stage.

YOLOv10 has the highest F1-score, precision, and accuracy on multispectral data, which proves its strength in the field conditions but with higher computational requirements. YOLOv8 remained competitive with comparable inference time and lower model complexity, making it more suitable for fast and real-time applications on resource-constrained hardware. The spectral response analysis affirms that the NIR and cyan bands are better at showing internal tissue damage in anthracnose, dieback, and sooty mould, whereas the RGB is better at showing the surface symptoms such as bacterial canker.

Due to its higher computational demand, YOLOv10 was less suitable for real-time embedded deployment. YOLOv8 was identified to be the most balanced option and one that is highly accurate, better at generalising to symptoms at early stages and can be used in edge hardware platforms. Additional performance improvements were achieved through the proposed Sequential SGD–AdamW (YOLOv8SO) optimisation strategy, which combined the convergence stability of SGD with a gradient efficiency of AdamW. This helped to minimise the training loss and stabilise inference performance.

The real-time implementation stage highlighted the feasibility of the proposed framework. YOLOv8SO models in ONNX format were deployed on both Raspberry Pi 5 and Jetson Orin Nano with the latter showing the best trade-off between speed and detection accuracy. In particular, it was demonstrated that inference at 1024 x 1024 resolution in ONNX format can be achieved with minor compromises in model or spectral fidelity and still be used to perform reliable in-field diagnostics. These findings place this framework as an asset to precision agriculture to aid in the early detection of diseases, protection of yield, and sustainable crop management.

Future work may focus on extending this framework by incorporating spectral vegetation indices such as NDVI or GNDVI. This may improve the sensitivity of the model to physiological stresses and disease severity. Moreover, incorporating a feedback loop with field-validated active learning strategies could allow the model to continually improve to accommodate seasonal, geographical, and disease variants changes. Future versions of this system could also be made to support microcontroller grade edge devices with the use of model compression and quantisation to increase accessibility further. Lastly, the inclusion of explainable AI (XAI) methods might provide the ability to make diseases predictions interpretable, and the stakeholders can see the results and reasoning behind the model decisions. To be deployed into a real-world, it would need other components like user interfaces, integration with farm management systems, as well as compliance with UAV user and safety regulations. The suggested scheme is not specific to the diseases of the mango leaf but may be expanded to other crops and disease varieties with the help of the same RGB and multispectral UAV sensors data.

The article provides a much-needed connection between high-performance deep learning models and real-time, field-deployable solutions. It contributes to the intersection of remote sensing, plant pathology, and embedded artificial intelligence for sustainable agricultural systems.

## Supporting information

S1 Table Architectural complexity and inference latency of YOLOv5-YOLOv10 models on RGB and multispectral data. (DOCX)

S2 Table Final deployment results on the Raspberry Pi 5 development board for YOLOv5-YOLOv10 using RGB and multispectral images resized to 1024 × 1024 pixels, evaluated with PyTorch (.pt) models. (DOCX)

S3 Table Final deployment results on the Raspberry Pi 5 development board for YOLOv5-YOLOv10 using RGB and multispectral images resized to 1024 × 1024 pixels, evaluated with ONNX models. (DOCX)

S4 Table Final deployment results on the NVIDIA Jetson Orin Nano development board for YOLOv5-YOLOv10 using RGB and multispectral images resized to 1024 × 1024 pixels, evaluated with PyTorch (.pt) models. (DOCX)

S5 Table Final deployment results on the NVIDIA Jetson Orin Nano development board for YOLOv5-YOLOv10 using RGB and multispectral images resized to 1024 × 1024 pixels, evaluated with ONNX models. (DOCX)
