# Supplementary material for: UAV-based RGB and multispectral mango leaf disease detection with benchmarking of YOLOv5 to YOLOv10 and SeqOpt-optimised YOLOv8 for real-time edge deployment
Source: PLoS One. 2026 May 28;21(5):e0349855. doi: 10.1371/journal.pone.0349855 (PMC13218508; doi:10.1371/journal.pone.0349855)
Supplement: S1 Table — (DOCX) [file pone.0349855.s001.docx]

**S1 Table Architectural complexity and inference latency of YOLOv5-YOLOv10 models on RGB and multispectral data.**

| **YOLO Version** | **Data Type** | **Layers** | **Parameters in Millions** | **GFLOPs** | **Inference time in milliseconds** |
| --- | --- | --- | --- | --- | --- |
|  |  |  |  |  |  |
| YOLOv5 | RGB | 193 | 9.11 | 23.8 | 18.9 |
|  | Multi | 193 | 9.11 | 23.8 | 27.7 |
| YOLOv6 | RGB | 372 | 20.11 | 116 | 12.2 |
|  | Multi | 372 | 20.11 | 116 | 12.4 |
| YOLOv7 | RGB | 208 | 6.02 | 13 | 5.7 |
|  | Multi | 208 | 6.02 | 13 | 5.8 |
| YOLOv8 | RGB | 168 | 11.13 | 28.4 | 24.3 |
|  | Multi | 168 | 11.13 | 28.4 | 24.1 |
| YOLOv9 | RGB | 486 | 7.17 | 26.7 | 30 |
|  | Multi | 486 | 7.17 | 26.7 | 26.5 |
| YOLOv10 | RGB | 293 | 8.04 | 24.5 | 21.2 |
|  | Multi | 293 | 8.04 | 24.5 | 19.7 |
| RGB and Multi denote RGB and multispectral OCN images, respectively. GFLOPs - Giga Floating Point Operations per Second. All experiments were conducted using the small (S) variants of each architecture. | | | | | |
