# Supplementary material for: UAV-based RGB and multispectral mango leaf disease detection with benchmarking of YOLOv5 to YOLOv10 and SeqOpt-optimised YOLOv8 for real-time edge deployment
Source: PLoS One. 2026 May 28;21(5):e0349855. doi: 10.1371/journal.pone.0349855 (PMC13218508; doi:10.1371/journal.pone.0349855)
Supplement: S2 Table — (DOCX) [file pone.0349855.s002.docx]

**S2 Table Final deployment results on the Raspberry Pi 5 development board for YOLOv5-YOLOv10 using RGB and multispectral images resized to 1024 × 1024 pixels, evaluated with PyTorch (.pt) models.**

| **YOLO Version** | **Data Type** | **Images count** | **P** | **R** | **mAP@50** | **mAP@ 50-95** | **F1** | **(Time(s)/ Image)/10** | **Energy /Image (Wh)*1000** |
| --- | --- | --- | --- | --- | --- | --- | --- | --- | --- |
| YOLOv10 | RGB | 977 | 0.936 | 0.798 | 0.88 | 0.756 | 0.862 | 0.4195 | 0.779939 |
|  | Multi | 818 | 0.947 | 0.829 | 0.905 | 0.802 | 0.884 | 0.4160 | 0.695599 |
| YOLOv9 | RGB | 977 | 0.926 | 0.677 | 0.814 | 0.669 | 0.782 | 0.4179 | 0.778915 |
|  | Multi | 818 | 0.925 | 0.736 | 0.852 | 0.721 | 0.82 | 0.4021 | 0.665037 |
| YOLOv8 | RGB | 977 | 0.94 | 0.743 | 0.852 | 0.697 | 0.83 | 0.3833 | 0.705220 |
|  | Multi | 818 | 0.934 | 0.782 | 0.878 | 0.748 | 0.851 | 0.3714 | 0.726161 |
| YOLOv8SO | Multi | 818 | 0.984 | 0.951 | 0.975 | 0.934 | 0.97 | 0.3334 | 0.718826 |
| YOLOv7 | RGB | 977 | 0.882 | 0.611 | 0.596 | 0.345 | 0.722 | 0.1281 | 0.398158 |
|  | Multi | 818 | 0.889 | 0.62 | 0.608 | 0.336 | 0.731 | 0.1303 | 0.323961 |
| YOLOv6 | RGB | 977 | 0.945 | 0.804 | 0.8 | 0.611 | 0.869 | 0.2363 | 0.738997 |
|  | Multi | 818 | 0.941 | 0.823 | 0.83 | 0.605 | 0.878 | 0.2350 | 0.649144 |
| YOLOv5 | RGB | 977 | 0.928 | 0.701 | 0.827 | 0.662 | 0.799 | 0.3440 | 0.632549 |
|  | Multi | 818 | 0.933 | 0.736 | 0.853 | 0.715 | 0.823 | 0.3396 | 0.562347 |
| Note: All results were obtained on the Raspberry Pi 5 using CPU-only inference with images resized to 1024 × 1024 pixels and PyTorch (.pt) model files. P - Precision, R - Recall, F1 - F1-score, and mAP denotes mean Average Precision evaluated at IoU thresholds of 0.5 (mAP@50) and 0.5-0.95 (mAP@50-95). Time (pre-process + inference + post-process) is reported in seconds (s) per image, and energy consumption is reported in watt-hours (Wh). Model abbreviations follow the convention: YOLOv8SO Multi refers to YOLOv8 with SeqOpt optimisation trained on multispectral data; RGB and Multi denote RGB and multispectral OCN images, respectively. All experiments were conducted using the small (S) variants of each architecture. | | | | | | | | | |
