# Supplementary material for: UAV-based RGB and multispectral mango leaf disease detection with benchmarking of YOLOv5 to YOLOv10 and SeqOpt-optimised YOLOv8 for real-time edge deployment
Source: PLoS One. 2026 May 28;21(5):e0349855. doi: 10.1371/journal.pone.0349855 (PMC13218508; doi:10.1371/journal.pone.0349855)
Supplement: S3 Table — (DOCX) [file pone.0349855.s003.docx]

**S3 Table Final deployment results on the Raspberry Pi 5 development board for YOLOv5-YOLOv10 using RGB and multispectral images resized to 1024 × 1024 pixels, evaluated with ONNX models.**

| **YOLO Version** | **Data Type** | **Images count** | **P** | **R** | **mAP@50** | **mAP@ 50-95** | **F1** | **(Time(s)/ Image)/10** | **Energy /Image (Wh)*1000** |
| --- | --- | --- | --- | --- | --- | --- | --- | --- | --- |
| YOLOv10 | RGB | 977 | 0.932 | 0.775 | 0.864 | 0.736 | 0.846 | 0.17655 | 0.495394 |
|  | Multi | 818 | 0.936 | 0.815 | 0.896 | 0.784 | 0.871 | 0.17536 | 0.460880 |
| YOLOv9 | RGB | 977 | 0.926 | 0.64 | 0.794 | 0.642 | 0.757 | 0.18322 | 0.593654 |
|  | Multi | 818 | 0.903 | 0.715 | 0.836 | 0.689 | 0.798 | 0.17536 | 0.500000 |
| YOLOv8 | RGB | 977 | 0.94 | 0.711 | 0.835 | 0.676 | 0.81 | 0.17116 | 0.568066 |
|  | Multi | 818 | 0.919 | 0.754 | 0.86 | 0.723 | 0.828 | 0.16086 | 0.591687 |
| YOLOv8SO | Multi | 818 | 0.986 | 0.95 | 0.974 | 0.923 | 0.968 | 0.12027 | 0.444988 |
| YOLOv5 | RGB | 977 | 0.908 | 0.682 | 0.81 | 0.634 | 0.779 | 0.17065 | 0.475947 |
|  | Multi | 818 | 0.921 | 0.709 | 0.838 | 0.691 | 0.801 | 0.16108 | 0.496333 |
| Note: All results were obtained on the Raspberry Pi 5 using CPU-only inference with images resized to 1024 × 1024 pixels and ONNX model files. P - Precision, R - Recall, F1 - F1-score, and mAP denotes mean Average Precision evaluated at IoU thresholds of 0.5 (mAP@50) and 0.5-0.95 (mAP@50-95). Time (pre-process + inference + post-process) is reported in seconds (s) per image, and energy consumption is reported in watt-hours (Wh). Model abbreviations follow the convention: YOLOv8SO Multi refers to YOLOv8 with SeqOpt optimisation trained on multispectral data; RGB and Multi denote RGB and multispectral OCN images, respectively. All experiments were conducted using the small (S) variants of each architecture. | | | | | | | | | |
