# Supplementary material for: UAV-based RGB and multispectral mango leaf disease detection with benchmarking of YOLOv5 to YOLOv10 and SeqOpt-optimised YOLOv8 for real-time edge deployment
Source: PLoS One. 2026 May 28;21(5):e0349855. doi: 10.1371/journal.pone.0349855 (PMC13218508; doi:10.1371/journal.pone.0349855)
Supplement: S4 Table — (DOCX) [file pone.0349855.s004.docx]

**S4 Table Final deployment results on the NVIDIA Jetson Orin Nano development board for YOLOv5-YOLOv10 using RGB and multispectral images resized to 1024 × 1024 pixels, evaluated with PyTorch (.pt) models.**

| **YOLO Version** | **Data Type** | **Images count** | **P** | **R** | **mAP@50** | **mAP@ 50-95** | **F1** | **(Time(s)/ Image) /100** | **(Energy(Wh) / Image) *10** |
| --- | --- | --- | --- | --- | --- | --- | --- | --- | --- |
| YOLOv10 | RGB | 977 | 0.936 | 0.798 | 0.88 | 0.756 | 0.862 | 0.000767 | 0.000348 |
|  | Multi | 818 | 0.946 | 0.829 | 0.905 | 0.802 | 0.884 | 0.000766 | 0.000330 |
| YOLOv9 | RGB | 977 | 0.926 | 0.677 | 0.814 | 0.668 | 0.782 | 0.000948 | 0.000409 |
|  | Multi | 818 | 0.924 | 0.736 | 0.852 | 0.721 | 0.819 | 0.000963 | 0.000379 |
| YOLOv8 | RGB | 977 | 0.936 | 0.798 | 0.88 | 0.756 | 0.862 | 0.000767 | 0.000338 |
|  | Multi | 818 | 0.946 | 0.829 | 0.905 | 0.802 | 0.884 | 0.000766 | 0.000296 |
| YOLOv8SO | Multi | 818 | 0.983 | 0.951 | 0.975 | 0.934 | 0.97 | 0.000720 | 0.000281 |
| YOLOv7 | RGB | 977 | 0.889 | 0.612 | 0.597 | 0.346 | 0.725 | 0.000507 | 0.000276 |
|  | Multi | 818 | 0.889 | 0.619 | 0.607 | 0.335 | 0.73 | 0.000505 | 0.000269 |
| YOLOv6 | RGB | 977 | 0.97 | 0.69 | 0.793 | 0.606 | 0.806 | 0.000826 | 0.000450 |
|  | Multi | 818 | 0.962 | 0.75 | 0.821 | 0.599 | 0.843 | 0.000809 | 0.000403 |
| YOLOv5 | RGB | 977 | 0.928 | 0.701 | 0.827 | 0.662 | 0.799 | 0.000693 | 0.000338 |
|  | Multi | 818 | 0.933 | 0.736 | 0.854 | 0.715 | 0.823 | 0.000711 | 0.000391 |
| Note: All results were obtained on the NVIDIA Jetson Orin Nano using GPU-accelerated inference with images resized to 1024 × 1024 pixels and PyTorch (.pt) model files. P - Precision, R - Recall, F1 - F1-score, and mAP denotes mean Average Precision evaluated at IoU thresholds of 0.5 (mAP@50) and 0.5-0.95 (mAP@50-95). Time (pre-process + inference + post-process) is reported in seconds (s) per image, and energy consumption is reported in watt-hours (Wh). Model abbreviations follow the convention: YOLOv8SO Multi refers to YOLOv8 with SeqOpt optimisation trained on multispectral data; RGB and Multi denote RGB and multispectral OCN images, respectively. All experiments were conducted using the small (S) variants of each architecture. | | | | | | | | | |
